# The DIOS framework for optimizing infectious disease surveillance: Numerical methods for simulation and multi-objective optimization of surveillance network architectures

**DOI:** 10.1371/journal.pcbi.1008477

**Published:** 2020-12-04

**Authors:** Qu Cheng, Philip A. Collender, Alexandra K. Heaney, Xintong Li, Rohini Dasan, Charles Li, Joseph A. Lewnard, Jonathan L. Zelner, Song Liang, Howard H. Chang, Lance A. Waller, Benjamin A. Lopman, Changhong Yang, Justin V. Remais

**Affiliations:** 1 Division of Environmental Health Sciences, School of Public Health, University of California, Berkeley, Berkeley, California, United States of America; 2 Department of Biostatistics and Bioinformatics, Rollins School of Public Health, Emory University, Atlanta, Georgia, United States of America; 3 Division of Epidemiology, School of Public Health, University of California, Berkeley, Berkeley, California, United States of America; 4 Department of Epidemiology, School of Public Health, University of Michigan, Ann Arbor, Michigan, United States of America; 5 Center for Social Epidemiology and Population Health, School of Public Health, University of Michigan School of Public Health, Ann Arbor, Michigan, United States of America; 6 Department of Environmental and Global Health, College of Public Health and Health Professions, and Emerging Pathogens Institute, University of Florida, Gainesville, Florida, United States of America; 7 Department of Epidemiology, Rollins School of Public Health, Emory University, Atlanta, Georgia, United States of America; 8 Institute of Health Informatics, Sichuan Center for Disease Control and Prevention, Chengdu, Sichuan, People’s Republic of China; Institute for Disease Modeling, UNITED STATES

## Abstract

Infectious disease surveillance systems provide vital data for guiding disease prevention and control policies, yet the formalization of methods to optimize surveillance networks has largely been overlooked. Decisions surrounding surveillance design parameters—such as the number and placement of surveillance sites, target populations, and case definitions—are often determined by expert opinion or deference to operational considerations, without formal analysis of the influence of design parameters on surveillance objectives. Here we propose a simulation framework to guide evidence-based surveillance network design to better achieve specific surveillance goals with limited resources. We define evidence-based surveillance design as an optimization problem, acknowledging the many operational constraints under which surveillance systems operate, the many dimensions of surveillance system design, the multiple and competing goals of surveillance, and the complex and dynamic nature of disease systems. We describe an analytical framework—the Disease Surveillance Informatics Optimization and Simulation (DIOS) framework—for the identification of optimal surveillance designs through mathematical representations of disease and surveillance processes, definition of objective functions, and numerical optimization. We then apply the framework to the problem of selecting candidate sites to expand an existing surveillance network under alternative objectives of: (1) improving spatial prediction of disease prevalence at unmonitored sites; or (2) estimating the observed effect of a risk factor on disease. Results of this demonstration illustrate how optimal designs are sensitive to both surveillance goals and the underlying spatial pattern of the target disease. The findings affirm the value of designing surveillance systems through quantitative and adaptive analysis of network characteristics and performance. The framework can be applied to the design of surveillance systems tailored to setting-specific disease transmission dynamics and surveillance needs, and can yield improved understanding of tradeoffs between network architectures.

## Author summary

Disease surveillance systems are essential for understanding the epidemiology of infectious diseases and improving population health. A well-designed surveillance system can achieve a high level of fidelity in estimates of interest (e.g., disease trends, risk factors) within its operational constraints. Currently, design parameters that define surveillance systems (e.g., number and placement of the surveillance sites, target populations, case definitions) are selected largely by expert opinion and practical considerations. Such an informal approach is less tenable when multiple aspects of surveillance design—or multiple surveillance objectives—need to be considered simultaneously, and are subject to resource or logistical constraints. Here we propose a framework to optimize surveillance system design given a set of defined surveillance objectives and a dynamical model of the disease system under study. The framework provides a platform for *in silico* surveillance system design, and allows the formulation of surveillance guidelines based on quantitative evidence, tailored to local realities and priorities. The framework is designed to facilitate greater collaboration between health planners and computational and data scientists to advance surveillance science and strengthen the architecture of surveillance networks.

This is a *PLOS Computational Biology* Methods paper.

## Introduction

Infectious disease surveillance systems provide vital information on patterns of disease occurrence across space, time, and populations of interest, and ultimately provide the basis for evidence-based disease control policy decisions [1]. Considerable progress has been made supporting infectious disease control decision-making with computational approaches to evaluate the outcomes of alternative decisions [2]. Examples include optimizing when, where, and among which populations to allocate public health resources [3,4], determining the optimal balance between multiple intervention approaches (e.g., case detection, treatment, vaccination, and sanitation improvement) [5–8], and optimizing the start time, duration, and dose of drug treatment programs [9,10]. In contrast, little attention has been paid to the development of tools for improving infectious disease surveillance system designs, and formalization of methods to optimize surveillance networks has largely been overlooked.

‘Design parameters’, which are high-level characteristics that define infectious disease surveillance networks—such as locations of surveillance sites, sampling frequency for laboratory testing or community-based surveys, and diagnostic techniques—can greatly influence the degree to which the resulting surveillance data serves public health objectives, including early detection of outbreaks [11,12], improved understanding of disease emergence and spread (e.g., emergence of a novel coronavirus disease in 2020) [13,14], and accurate measurement of the impact of interventions [15]. Thus, key design parameters can be modified in a manner informed by optimization analysis such that the system better achieves specific surveillance goals. Examples of surveillance design optimization include relocating and adding reporting sites to predict the temporal trend of diseases more accurately [16,17]; changing diagnostic approaches/case definitions to increase the chance of detecting cases [18]; and targeted sampling of specific subpopulations to improve the timeliness of outbreak detection [19–21].

In practice, surveillance system design parameters are often set in an *ad hoc* fashion based on operational considerations (e.g., budget, convenience, political agendas), rather than through quantitative evaluation of how alternative designs might impact surveillance system objectives. For instance, World Health Organization (WHO) recommends selection of influenza surveillance sites based on the facilities’ willingness to participate, availability of necessary laboratory and information infrastructure, ability to cover the surveillance cost, and representativeness of the general population. Notably absent from these criteria is the degree to which the network’s performance on specific surveillance objectives will be enhanced [15]. The absence of objective criteria and methods to evaluate and iteratively reconfigure surveillance system design can lead to inefficient use of limited resources. For example, in China, current requirements specify that 5–15 influenza-like illness (ILI) cases are required to be sampled per week at each of the 556 influenza sentinel hospitals for laboratory confirmation [22]. If the total sample size is fixed, it may be that reducing the number of sentinel sites (e.g., prioritizing sites in populous regions and with high levels of population movement), while increasing the number of samples collected at each site, could yield more timely detection of outbreaks with the same level of resources. What is more, because disease surveillance systems generally operate in pursuit of multiple objectives, optimal design can be highly counterintuitive.

Recent research has provided some initial examples of quantitative infectious disease surveillance design optimization [23,24]. In one study, researchers estimated that an optimal relocation of Iowa’s existing 22 ILINet sentinel sites could increase population coverage of the network from 56% to 75% [25]. In another example, targeted surveillance of pregnant women was estimated to increase the weekly probability of detecting Zika virus introduction from 11% to 40%, in comparison with surveillance of blood donors [18]. While these and other studies serve as foundational examples, the methods utilized in these analyses are targeted towards narrow, study-specific objectives and specific networks, and are challenging to generalize to other—even closely related—surveillance design optimization problems. What is more, prior studies have not attempted to articulate a general theory of surveillance design optimization and decision-making.

### Surveillance design as a multi-objective, multi-dimensional, constrained and dynamic optimization problem

The search for optimal disease surveillance designs is a highly complex problem due to multiple, often competing goals of surveillance data collection, idiosyncratic surveillance network design, operational constraints that govern surveillance systems, and the complexity and dynamic nature of diseases under surveillance. Simple optimization problems involving a single objective and limited possible designs—such as the optimal placement of a new surveillance site among 100 alternatives in order to maximize the proportion of influenza cases detected—may be solved in relatively straightforward fashion by testing all possible designs and choosing the design that results in optimal performance (e.g., the new site location that results in the highest proportion of cases detected overall). However, surveillance network optimization quickly becomes non-trivial when the design space expands (e.g., selecting 10 sites out of 200 alternative sites), when multiple objectives (such as increasing case detection, improving spatial and temporal trend estimation, and risk factor identification) are subject to simultaneous analysis and optimization, or when optimization is subject to constraints regarding resource limitations and operational plausibility. Uncertainty regarding the functioning of the epidemiologic system and shifts in patterns of diseases further complicate matters. Hence, our optimization goals are multidimensional, dynamic, and stochastic. In this section, we describe the relevance of surveillance objectives, network design parameters, operational constraints and dynamic disease systems to the pursuit of surveillance optimization.

#### Multiple objectives

Disease surveillance systems are established and designed for diverse purposes, including to collect data for understanding variations in disease frequency across populations, space, and time, to monitor pathogen composition over time, to detect outbreaks and forecast epidemics, to assess the impact of interventions, and to determine risk factors associated with diseases. Most surveillance systems operate with multiple public health objectives. Hence, surveillance system designs should generally be subject to multi-objective optimization, and tradeoffs between different objectives must be considered. For instance, if the goals of a system are to both estimate prevalence and assess the impact of risk factors, the network design should be subjected to optimization routines capable of capturing tradeoffs between designs with respect to achieving these two objectives. Input from public health officials can help identify surveillance needs and goals and verify the degree to which the formulated objective functions are able to express the performance of the surveillance system on these goals. Moreover, the DIOS framework can be adapted for optimizing surveillance systems that target multiple infections simultaneously, including diseases with shared etiologies such as those monitored by the U.S. ILINet system (respiratory infections including influenza virus, respiratory syncytial virus, human coronavirus, adenovirus, etc.) [26,27]. To accomplish this, the disease system and surveillance system models could include microbiologically nonspecific outcomes (e.g., acute respiratory illness, acute gastroenteritis, etc.), as well as subroutines to simulate each specific infection of interest. Objective functions could encompass goals defined for the overall family of diseases (e.g., monitoring total respiratory disease), as well as for its specific members (e.g., monitoring influenza). Importantly, for groups of diseases sharing similar transmission characteristics, common risk factors and key interactions between pathogens—such as clinical presentations, competition for host resources, induced changes in host immunity, or competitive inhibition—can be exploited to yield more efficient use of multi-disease data and therefore more efficient surveillance designs. Taking the example of a DIOS application to a cluster of seasonal respiratory diseases, the disease model might be structured as a set of coupled spatio-temporal models capturing the dynamics of each specific respiratory infection, as well as interaction between them using shared spatial and temporal random effects (following prior work using a shared component approach [28–30]). Such coupled models are able to borrow information across diseases, potentially requiring less sampling effort to achieve the desired surveillance performance.

#### Multiple design parameters

Surveillance system structure and design can be decomposed into a multitude of characteristics, operational details, and features that influence the performance of surveillance networks. These design parameters and their impacts on system performance can then be represented and simulated within models. For example, to improve estimation of disease incidence, either the accuracy of diagnostics at existing reporting facilities or the number of facilities in the reporting network, or both, can be modified. Other design parameters, such as when, where, and among which populations to implement targeted sampling efforts may also be entered into the analysis, greatly expanding the dimensionality of the problem. Moreover, the set of design parameters to optimize depends on the surveillance goals. For example, when the surveillance goal is accurate estimation of the temporal trend of a disease, it may be that the placement of sites is less important than sampling frequency. Professional users within the public health community can provide guidance on which design parameters are modifiable, and which may—upon modification—yield improved surveillance performance on these predefined surveillance goals. Table 1 lists examples of design parameters, their potential impacts on surveillance system performance, and their occurrence in real world infectious diseases surveillance systems.

**Table 1:** Example surveillance system design parameters and their potential impacts on surveillance performance.

Design parameter	Definition	Potential impacts on surveillance performance	Example designs	Example surveillance system	Ref.
**Target population**	Population to be monitored for disease outcomes of interest	Target populations representative of a general population provide a means of tracking overall disease incidence and trends in the population as a whole. Target populations informed by demographic differences in disease risk, transmission potential, or detection probability may provide advantages for monitoring outcomes in vulnerable populations, anticipating outbreaks, or tracking rare diseases	All persons >2 years of age residing in homes	Republic of South Africa HIV prevalence survey	[31]
			Pregnant women and infants	US Zika Pregnancy and Infant Registry	[32]
**Site enrollment**	The inclusion of hospitals and other facilities in passive reporting networks, or selection of locations for active surveillance	Site selection influences factors such as population coverage and representativeness, diagnostic quality, the speed at which spreading outbreaks may be detected, and informational redundancy due to spatial proximity or other sources of similarity between locations	Hospitals in Maluku, North Sulawesi, East Kalimantan, North Sumatra, Yogyakarta and West Nusa Tenggara	Indonesia influenza sentinel surveillance system	[33]
			Health centers in Dembi, Asendabo, Tulubolo, Guangua, Bulbula, Dhera, Welenchity, Metahara, Asebot, and Kersa	Ethiopia malaria sentinel surveillance	[34]
**Sampling strategy**	Type of sampling used to identify cases among the target population	Sampling strategies influence the representativeness of surveillance data, as well as the ability of surveillance systems to detect rare or underreported conditions. Strategies that adequately characterize a general population may be biased with respect to critical subpopulations	Hospital-based convenience sampling (e.g., every fourth patient meeting case definition)	Bangladesh rotavirus surveillance system	[35]
			Respondent-driven sampling, which uses existing samples in high-risk groups (e.g., intravenous drug user, men who have sex with men) to recruit new samples, then uses a model to correct for potential bias in the nonprobability sampling	Central America sexual behaviors and HIV prevalence survey	[36]
**Sampling intensity**	Number of samples per sampling interval	Under operational constraints, the choice between sampling more frequently but with low intensity or less frequently with higher intensity represents a tradeoff between the ability to resolve high frequency changes in outcomes of interest, or timeliness of detection, and reducing statistical uncertainty	3 adults and 2 children per week	Malaysia laboratory-based influenza surveillance system	[37]
			5 mild cases serotyped per month per site	China hand, foot, and mouth disease sentinel surveillance system	[38]
**Sampling seasonality**	Pre-determined changes in sampling intensity over time	Year-round sampling increases the chances of detecting unexpected changes in disease incidence. However, if disease seasonality is static and well-understood, resources may be better used for intensive seasonal sampling	Year-round	New Zealand virological surveillance system	[39]
			Transmission season (June-October)	China dengue virological surveillance system	[40]
**Laboratory diagnostics**	Methods used to determine the presence of a pathogen	Diagnostic tests and other related factors such as specimen types, the quality of the specimen, and the time from onset to specimen collection can influence the sensitivity and specificity of the surveillance system	Isolation of *Bordetella pertussis* from clinical specimen and/or a four-fold or greater increase in titer of antibody against *B*. *pertussis* between acute and convalescent sera	China pertussis surveillance system	[41]
			Isolation of *B*. *pertussis* from clinical specimen and positive polymerase chain reaction (PCR) for *B*. *pertussis*	US CDC pertussis surveillance system	[42]
**Case definition**	Diagnostic criteria to classify outcomes of interest	Case definitions can influence factors such as the severity and characteristics of cases identified, and the sensitivity and specificity of the system	Influenza-like illness, defined as an acute respiratory infection with measured fever of ≥ 38°C and cough with onset within the last 10 days	WHO global influenza surveillance	[43]
			Severe acute respiratory infection, defined as an acute respiratory infection with history of fever or measured fever of ≥ 38°C and cough with onset within the last 10 days and requires hospitalization		

#### Operational constraints

Operational restrictions on surveillance system designs—due to budgetary, logistical, political and cultural considerations—add critical constraints to the optimization problem. Absent constraints, the optimal design may be self-evident, e.g., sampling at maximal frequency and intensity. Yet when there is a fixed budget for samples, tradeoffs arise between plausible designs and competing objectives. For example, the optimal balance between design parameters—say, number of samples and sampling frequency—depends on the relative value of precise cross-sectional estimates of disease prevalence versus characterizing disease incidence over time, which in turn depends on the specific objectives of surveillance and the dynamics of the underlying disease system. Public health officials can help set these operational constraints using information on available resources, logistics and political considerations for the surveillance system of interest.

#### Dynamic and imperfectly understood disease systems

Surveillance systems must respond to shifts in the epidemiology of target infections. As infections emerge, become endemic, or approach elimination within populations or subpopulations, and as the state of knowledge on the target disease systems evolves, the goals of surveillance, and the resulting optimal designs, can (and must) evolve alongside them. The dynamic nature of optimal surveillance design may be especially important in developing economies that are undergoing epidemiologic transitions. For instance, as a region or nation approaches elimination of a particular infectious disease, surveillance goals generally shift from enumeration of endemic cases occurring in the general population to detection of nexuses of sporadic transmission. This may require new designs (e.g., shifting to more intensive surveillance within a limited area, or increasing the coverage of subpopulations involved in ongoing transmission), and adjustment of system objectives (e.g., maximize detection of the few remaining cases instead of optimizing estimates of incidence in the general population). Conversely, as cases caused by novel pandemics (e.g., the 2020 coronavirus disease pandemic, or 2009 H1N1 pandemic) start to increase exponentially, surveillance systems may need to switch from tracking individual cases to population-based surveillance (e.g., performing laboratory testing among a proportion of patients with a non-specific syndrome) in order to monitor the progression of the outbreak and develop mitigation strategies without depleting public health resources.

When a disease system is poorly understood, as in the case of a novel emerging disease (e.g., Coronavirus Disease 2019, COVID-19), optimal surveillance system designs are likely to be subject to considerable epistemic uncertainty. This uncertainty can be represented within the DIOS framework by using an ensemble of plausible disease system models to simulate epidemiologic states during optimization, such models may be mechanistic or phenomenological with different structures in nature depending on the state of knowledge and operational questions to be addressed. The resulting variance in surveillance performance (i.e., objective function values) across the design space may result in identification of a large set of designs that are not significantly Pareto dominated on one or more objectives, but it may still be possible to exclude large under-performing regions of design space. As knowledge of the target disease evolves, the ensemble of disease system models can be refined by updating weights for different model structures or adjusting parameter distributions, and optimal designs can be re-evaluated.

Here, we present for the first time a unified analytical framework for quantitative infectious disease surveillance system optimization, accommodating multiple surveillance design parameters, objectives, operational constraints, and underlying disease processes. A common framework and standard terminology can enable closer collaboration between and among computational researchers, public health officials, clinical care providers and laboratories, and other stakeholders regarding the design and implementation of infectious disease surveillance systems. This in turn can accelerate the pace of methodological innovations and facilitate the development of surveillance design theories that anticipate and respond to current and future epidemiological challenges. Furthermore, a generalized framework can inspire the application of quantitative surveillance optimization across broader settings, resulting in system designs better aligned with local realities and public health priorities.

## Design and implementation

### The DIOS framework for surveillance simulation and optimization

The aforementioned challenges of surveillance optimization—multiple objectives, complexity of relevant design parameters, operational constraints, and dynamic and uncertain epidemiology of target diseases—suggest the need for a formalized framework for surveillance network optimization. Advances in computation for simulation-based studies have benefitted many related fields, including optimal disease control [44–47], yet applications of simulation optimization to the design of disease surveillance networks have scarcely been pursued. In the following sections, we detail the Disease Surveillance Informatics Optimization and Simulation (DIOS) framework, a simulation and optimization platform for designing infectious disease surveillance networks, and we demonstrate its application in a site selection context. DIOS facilitates a quantitative approach to designing surveillance systems tailored to local disease transmission dynamics and surveillance needs, as well as a more general study of optimal network design principles under varying objectives and epidemiological circumstances. Box 1 provides illustrative examples of potential DIOS applications.

**Box 1. Example DIOS applications.**

Text colors highlight the components of each optimization problem: design parameters (green), surveillance objectives (orange), and operational constraints (blue).

- What is thedistributionofsamplingeffortacrossagegroups that minimizesthetimetodetectionofinfluenzaoutbreaks? (One design parameter and one objective)
- Howmanytestkitsshouldbeallocatedtoeachcounty in order to minimizethenumberofcases and deaths caused by COVID-19, when onlyafixednumberoftestkitsareavailableperday? (One design parameter and multiple objectives)
- What is the distributionofsamplingeffort across multipletimepoints that minimizesabsoluteerrorinestimatedseasonality of hand, foot, and mouth disease, when thetotalsamplesizeperyearisfixed? (Multiple design parameters and one objective)
- When an endemic disease is nearing elimination (e.g., malaria), when and where should active sampling efforts be directed to minimizethetimetoeffectiveisolationandtreatmentofcases, as well as theprobabilityofre-establishmentofinfectioninpreviouslyclearedareas? (Multiple design parameters and multiple objectives)

**Fig 1:**
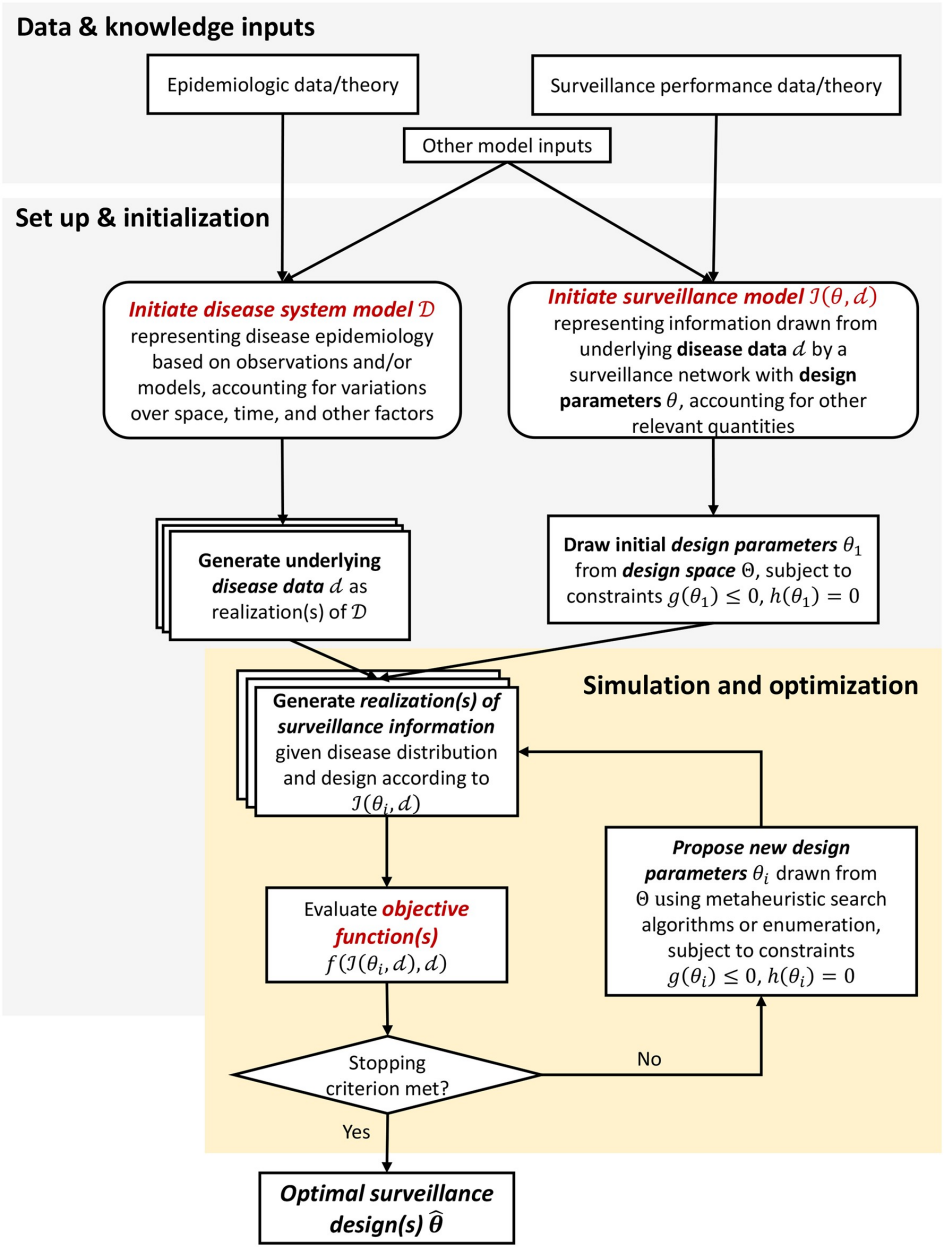
Schematic of the DIOS framework. The surveillance system optimization procedure uses data and knowledge about disease transmission and case ascertainment to identify optimal surveillance designs with regard to predefined surveillance goals. First, a disease system model D is defined, using observed epidemiologic data and/or theory, and taking into account relevant factors influencing disease dynamics or distribution. Multiple realizations of disease data (d) may be generated to explore optimal designs under uncertainty or variability of the underlying system (see *Specify and parameterize disease system model*). Furthermore, an ensemble of disease models can be combined to reduce the chance of model misspecification. Next, a surveillance model is defined to represent how information on the state of the disease system is captured as a function of design parameters *θ* and any other relevant variables (e.g., factors known to affect the sensitivity and specificity of a diagnostic test, or estimated underreporting rates for an area; see *Specify and parameterize surveillance model*). To initiate the optimization process, an initial design parameter set, *θ*_1_, is drawn from the design space subject to operational constraints *g*(*θ*_*i*_) ≤ 0, *h*(*θ*_*i*_) = 0 and, along with underlying disease data d, input to the surveillance model to generate a realization of surveillance information, $I_{1}=I(\theta_{1},\;d)$. The objective function, *f*, is evaluated based on the disease data d, and surveillance information $I_{1}$ (see *Define objective function(s)*). If a stopping criterion (e.g., reaching a large number of iterations; *de minimis* improvement in objective function) is not met, a new design parameter set, *θ*_*i*_, is proposed from the design space using metaheuristic search algorithms (e.g., simulated annealing, genetic algorithm, particle swarm algorithm) when the design space is large, or enumeration when the design space is small. This new design parameter set is then used to generate a new realization of surveillance information and evaluation of the objective functions (see *Simulation optimization search*). After a stopping criterion is met, design parameter sets with the best objective function values are output as optimal surveillance designs.

Broadly, the DIOS framework (Fig 1) allows for evaluation of surveillance system performance across a predefined design space under different epidemiologic scenarios (disease system model) and surveillance network characteristics (surveillance model). Numerical optimization algorithms (simulation optimization search) are applied to efficiently identify the region(s) of design space that yield superior network performance based on one or more specific surveillance goals. The optimization procedure (Fig 1 and Box 2) yields a set of network designs (i.e., optimal design parameter values) that maximize performance with respect to the specified public health goal(s), according to the specified data and models.

**Box 2. Surveillance system optimization procedure.**

**Input**: Epidemiologic data and/or theory, surveillance performance data and/or theory, and other auxiliary data (e.g., disease risk factors)

**Output**: the design parameter set with the highest/lowest (i.e., optimal) objective function value

**Initialization**:

 Define a disease system model to represent the underlying dynamics of the target disease system in the spatial, temporal, and demographic context of interest

 Generate disease distributions d as realization(s) of the system

 Sample initial design parameter set, *θ*_1_, within the design space subject to constraints *g*(*θ*_*i*_) ≤ 0, *h*(*θ*_*i*_) = 0

 Generate realization(s) of surveillance information,$I_{1}$, given d and *θ*_1_

 Evaluate objective function(s) *f* given $I_{1}$ and d

**while** stop criterion is not met **do**

 Propose a new design parameter set, *θ*_*i*_, within the design space using metaheuristic search algorithms or enumeration

 Generate realization(s) of surveillance information, $I_{i}$, given d and *θ*_*i*_

 Evaluate objective function(s), *f*, given $I_{i}$ and d

**end while**

**return** the best design parameter set, $\hat{\theta }$ (i.e., with the optimal objective function value)

### Specify and parameterize disease system model

An accurate representation of epidemiologic characteristics of the target disease(s) is essential for a successful optimization. This representation can be generated using observational data, outputs of mechanistic transmission models, or other approaches, and represents the best estimate of the disease’s epidemiology that is used to evaluate surveillance network performance using objective functions (see *Define objective function(s)*). To contend with potential model-misspecification and stochastic uncertainty, multiple realizations from an ensemble of disease models (i.e., with varying epidemiologic parameter values or different model structures) can be utilized in the framework. The structure of the disease system model output—such as spatial and temporal resolution—should be tailored to the surveillance objectives and design parameters. For instance, if a surveillance objective is to better estimate the spatial distribution of a disease, the target disease data must include geographical information about cases. If there are multiple target diseases of interest, disease models can be structured so as to represent the dynamics or distribution of the cluster of target diseases.

### Specify and parameterize surveillance model

In order to identify optimal network designs, a model representing key aspects of the sampling of and extraction of information from underlying disease processes by the surveillance system is needed. The surveillance model represents the mechanisms through which variation in network design parameters are expected to impact the epidemiologic information obtained and thus directly influences optimization with respect to surveillance objectives. Surveillance models generally comprise a set of probability distributions relating target estimands to the underlying disease state of the system, conditional on network design and other relevant considerations. For example, to optimize diagnostic protocol for minimal bias in reporting, a surveillance model may be constructed for the distribution of reported cases conditioned on diagnostic method, prevalence of the target disease relative to conditions with similar clinical presentation, and the distribution across subpopulations of factors that impact diagnostic sensitivity and specificity. When random errors contributed by surveillance processes are not explicitly taken into account, as may be the case when seeking to maximize the size of the population covered by a surveillance network, the surveillance model becomes a set of conditional Dirac delta distributions, and is deterministic. During the process of surveillance model specification, aspects of surveillance design that will be allowed to vary during optimization (i.e., the parameters to be optimized), and those that will be fixed (i.e., design aspects that are relevant to performance, but which it is not feasible or desirable to change) must be decided upon. Surveillance models may be as granular (e.g., modeling the full sequence of events necessary for each individual case to be reported) or abstract (e.g., modeling the overall proportion of cases detected in a population) as is deemed necessary for the optimization procedure, recognizing, however, that computational complexity may limit the feasibility of certain representations.

### Define objective function(s)

Changes to design parameters can be analyzed in relation to their influence on network performance in the context of specific surveillance system objectives. That is, performance is evaluated with respect to achieving a specific goal or goals. This evaluation is formalized by defining objective functions, which define the specific minimization or maximization problem to be solved, based on the design parameters and surveillance goals of interest. Thus, network performance is estimated through the iterative evaluation of objective functions, which are minimized (or maximized) as the design parameter space is searched. Table 2 presents canonical objective functions available for use in surveillance network optimization. Our examples do not explicitly include operational considerations within objective functions, but these can easily be taken into account. For example, the objective function could be established so as to yield the marginal information gain per added site or sample, or per dollar spent on surveillance. For a multi-disease optimization problem, objective functions can be defined to represent measures of performance of the system across all monitored diseases, as well as measures of performance for each disease individually. For example, the mean absolute errors in incidence of all diseases monitored by the surveillance system or the impact of surveillance on a common outcome (e.g., hospitalizations averted via case detection and isolation) could be used as the objective for a single-objective optimization, while disease-specific mean absolute errors could be added in a multi-objective optimization.

**Table 2:** Examples of objective functions for optimization analysis of surveillance networks.

Objective function type	Description	Example objective functions
Minimize mean error magnitudes	On average, how different a quantity, Q_I_, measured or estimated from the ascertained data I(θ, d), is from the same quantity, Q_D_, estimated or measured from the underlying disease data *D*. Includes mean squared error, mean squared percentage error, root mean squared error, standardized root mean squared error, mean absolute error, mean absolute percentage error, or other expressions.	To better ***characterize geographic*, *temporal*, *or demographic distribution of disease***, the objective function may be expressed as: $f=\sum_{i=1}^{n}{\left(C_{I,i}-\;C_{D,i}\right)}^{2}/n$ *n*—number of subpopulations *C*_*I*,*i*_—number of ascertained cases in subpopulation i *C*_*D*,*i*_—number of true cases from *D* in subpopulation i
		To ***assess the impact of interventions*** more accurately, the objective function may be expressed as: $f=|\frac{D_{I}}{F_{I}}-\frac{D_{D}}{F_{D}}|$ *D*_*I*_—number of cases detected by surveillance system in presence of intervention *F*_*I*_—number of cases detected by surveillance system in absence of intervention *D*_*D*_—true number of cases in presence of intervention *F*_*D*_—true number of cases in absence of intervention
Minimize uncertainty of surveillance estimands	If bias in surveillance sampling and estimation is not a concern (e.g. for asymptotically unbiased estimators), then minimizing uncertainty may be the primary goal. Uncertainty can be represented by standard error, standard deviation, inter-quantile range, or other expressions.	To determine the ***effect of a risk factor on infection*** more precisely when assuming a linear relationship between the risk factor and disease rate, the objective function may be expressed as: $f=\text{var}({\hat{\beta }}_{I})$ ${\hat{\beta }}_{I}$—estimated regression coefficient of the effect of the risk factor on the disease rate from the ascertained data
		To ***forecast the peak case count*** more precisely, the objective function may be expressed as: $f=\text{var}(P_{I})$ *P*_*I*_—forecasted peak case count based on ascertained data overall or for a specific area
Maximize log-likelihood	If a probability distribution *Q*_*I*_ ~ *Q*(*θ*, …) can be expressed by the surveillance model, then maximizing the likelihood of true data *Q*_D_ under the estimated distribution can be used to simultaneously address bias and variance.	To better estimate the ***effect of a risk factor on infection rates*** when assuming a linear relationship between the risk factor and disease rate, the objective function may be expressed as: $f=\text{log}(\frac{1}{\sigma \sqrt{2\pi }}e^{-\frac{1}{2}{\left(\frac{{\hat{\beta }}_{I}-\beta_{D}\;}{\sigma }\right)}^{2}}),$ if a normal distribution with a variance of σ^2^ is assumed for the true effect of a risk factor *β*_*D*_
		To ***improve estimation of outbreak probabilities***, the objective function may be expressed as: $f=\sum_{t=1}^{T}{[Y}_{t}\text{log}\left({\hat{p}}_{t}\right)+(1-Y_{t})\;\text{log}\left(1-{\hat{p}}_{t})\right],$ if outbreak probabilities in subsequent weeks are assumed to be conditionally independent. ${\hat{p}}_{t}$—estimated outbreak probability in time period *t* *Y*_*t*_—indicator (0 or 1) for actual occurrence of an outbreak in time period *t*
Maximize classification performance	When Q_I_ and Q_D_ are categorical, the performance of the surveillance system can be measured by classification evaluation metrics, such as sensitivity, specificity, positive predictive value, F1 scores, area under the receiver operating characteristic curve, etc.	To improve our ability to ***discriminate outbreaks from false alarms***, the objective function may be expressed as the area under the ROC curve: $f=\int_{0}^{1}\pi_{tp}(\pi_{fp})d\pi_{fp}$ *π*_*tp*_—proportion of true outbreaks correctly identified *π*_*fp*_—proportion of non-outbreak time periods falsely identified as outbreaks
		To improve our ability to ***detect a rare disease***, the objective function may be expressed as the maximum of the average *F*_1_ score: $f=2\int_{0}^{1}\frac{\pi_{tp|p}(\tau )\;\times \pi_{tp}(\tau )}{\pi_{tp|p}(\tau )+\pi_{tp}(\tau )}\;d\tau$ *π*_*tp*_—proportion of true cases reported *π*_*tp|p*_—proportion of reported cases that are true *τ*—threshold condition for reporting a case, assumed in this example to represent a probability

### Simulation optimization search

The goal of the optimization process (*while* block in Box 2; the loop in *Simulation optimization search* component of Fig 1) is to thoroughly explore the response surface of the objective function(s) over the design space so as to identify designs likely to yield optimal or near-optimal surveillance performance. Candidate surveillance designs are drawn from the design space, and the expectations of resulting objective function values across realizations are evaluated by comparing information ascertained by the surveillance system to the true underlying disease data; this process is repeated iteratively until a stopping criterion is reached, e.g., convergence on an estimated optimum; exhaustive sampling of the design space; or the exceedance of a computational budget. When the design parameter space is small, exhaustive evaluation of objective function values across the entire design parameter space may be feasible. Sufficient and efficient searching of large design parameter spaces, by contrast, may require heuristic or metaheuristic optimization algorithms (e.g., simulated annealing, genetic algorithms, particle swarm optimization, or Bayesian model-based optimization).

Multiple surveillance objectives can be optimized simultaneously through multi-objective optimization approaches, such as through weighted sums of objective functions or Pareto optimization [48]. Generating weighted sums of objective function values allows for the specification of relative importance of different objectives. If one objective is less important, it would be assigned a smaller weight when compared with other objectives and contribute less strongly to the identification of optimal designs. Pareto optimization outputs a set of optimal solutions (Pareto optimal set) for which no other solutions can perform better under all objectives. That is, improving the performance on one objective leads to worsening at least one of the other objectives. Decision makers are then tasked with choosing the “best” design from the Pareto optimal set by considering the relative importance of each objective, or other considerations not explicitly accounted for during optimization. Multi-objective optimization in the presence of a large design space can be handled by modified metaheuristic algorithms [49]. For example, to accommodate multiple objectives, Pareto simulated annealing approaches seek to express the acceptance probability of a new design as a function of its improvements in all objectives when compared with the current best design [50].

## Results

### Demonstration of the DIOS framework: Optimal selection of new surveillance sites

Here, we demonstrate an application of the DIOS framework in the context of selecting candidate sites to add to an existing cross-sectional survey network. We note that is but one potential application of the framework, and that the full set of surveillance design problems to which DIOS can be applied is vast, including establishing optimal temporal sampling regimes, targeted surveillance of important subpopulations, determination of optimal diagnostic criteria, and many others (Table 1). We consider two surveillance design objectives in this demonstration: (1) optimal prediction of the geographical distribution of the disease (hereafter referred to as *spatial prediction*); and (2) optimal estimation of the effect of a risk factor (hereafter referred to as *effect estimation*). We demonstrate how optimal designs can vary in relation to epidemiological characteristics of the target disease; in this case, we consider the rate of decrease in correlation of disease prevalence rates over distance, which determines whether prevalence changes abruptly or smoothly over the spatial domain. The simplified formulation of the site selection problem presented here is meant to be demonstrative of the general capabilities of DIOS, rather than as a comprehensive treatment of site selection applications of the framework. The code for the demonstration is available at https://github.com/OPTI-SURVEIL/DIOS_demonstration/.

We first describe the demonstration setting, the data available for design optimization, the specification and parameterization of the disease and surveillance system models, and the resulting formalized objective functions for optimizing spatial predictions and effect estimation. We demonstrate the use of an exhaustive search strategy to find the single most optimal site to add to the existing network for both goals, as well as the Pareto-optimal set of single sites to add when considering both objectives simultaneously. We simulate the addition of an arbitrary number of sites, acknowledging that in real-world applications of DIOS, the number of sites might be determined by budgetary constraints and/or the marginal informational gains per added site. We conclude our demonstration by considering the best set of three sites to add, which introduces substantial combinatorial complexity, motivating the use of a metaheuristic algorithm to efficiently search for optimal regions of design space.

### Demonstration setting

**Fig 2:**
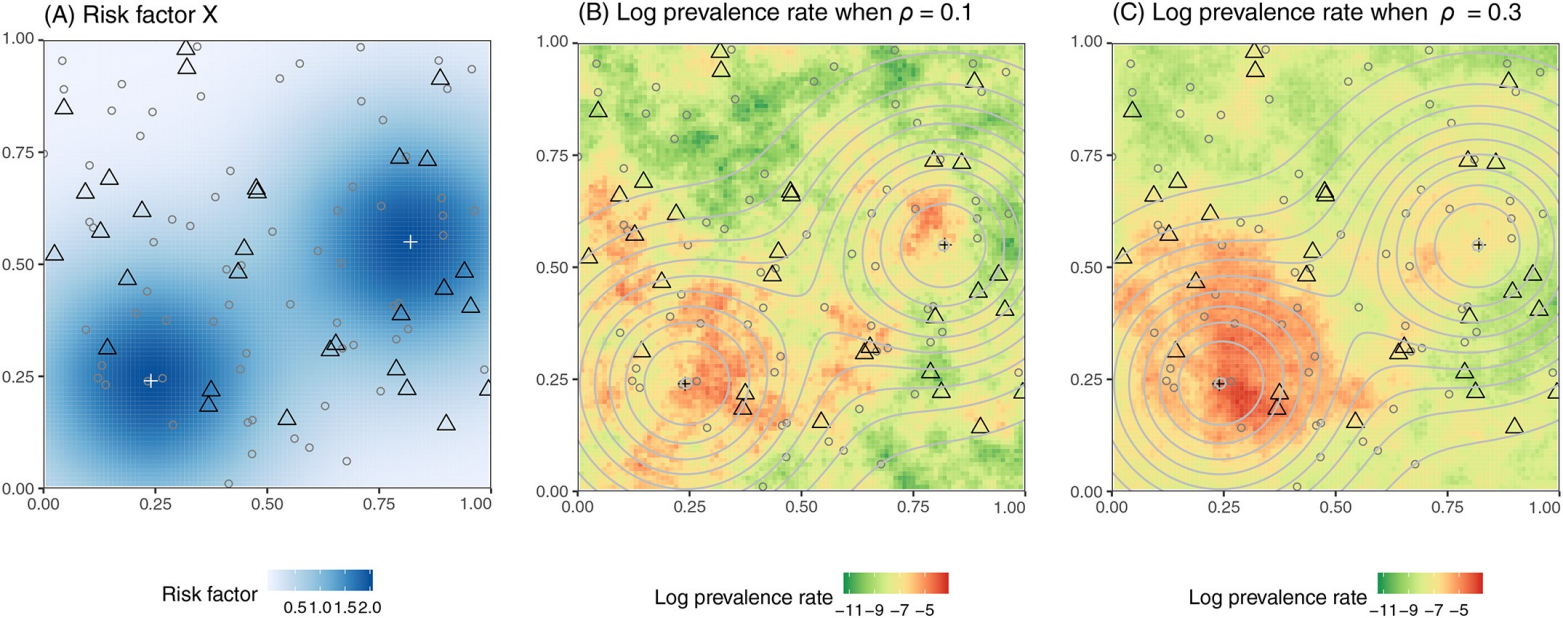
Simulated data used for surveillance system optimization. Spatial variation of (A) the risk factor *X* and (B) log prevalence when *ρ* = 0.1 and (C) *ρ* = 0.3. Triangles represent the existing 30 surveyed sites; dots represent the 70 candidate sites; crosses represent two point sources of the risk factor of interest (e.g. locations of mass gatherings); background color in Panel A and contour lines in panels B and C represent the levels of risk factor *X*. Three realizations of the log prevalence surface when *ρ* = 0.1 or 0.3 are shown in S1 Fig.

We generated a set of 100 potential surveillance sites scattered uniformly at random across a unit grid, and randomly selected 30 sites to represent a virtual existing surveillance network. We seeded two point sources for a risk factor influencing expected disease prevalence rates (Fig 2A), then simulated disease prevalence under two scenarios of spatial auto-correlation by adjusting the scale parameter (*ρ*) of a log-Gaussian spatial process centered on a linear function of the risk factor. We refer to these as spatially patchy (*ρ* = 0.1; Fig 2B) and spatially smooth (*ρ* = 0.3; Fig 2C) disease scenarios. Additional details of data generation are provided in S1 Text.

### Data and knowledge inputs

Available epidemiologic data to characterize the relevant aspects of the disease system include simulated prevalence rates observed at the 30 sites enrolled in the surveillance network. Data characterizing the surveillance system and design space include the coordinates of the 30 enrolled and 70 candidate sites. Additional data to support optimization include levels of risk factor *X* at each sampling location. Theoretical inputs include the assumption of a log-linear relationship between *X* and disease prevalence, and that spatial disease prevalence residuals follow a Gaussian process with exponential covariance function.

### Set up and initialization

#### Disease system model specification and simulation

In this demonstration, relevant aspects of the disease system include the correlation of disease outcomes over space, as well as the association of disease outcomes with risk factor *X*. Based on the observed disease prevalence at participating sites, we assume the log of the prevalence, *Y*, is generated from an underlying random spatial process with an independent and identically distributed (*i*.*i*.*d*) mean-zero normally distributed noise *ε* with a variance of *σ*_*d*_^*2*^, and can be modelled by:

$$Y=\text{exp}\left(\beta_{0}+\beta_{1}X+\eta +\varepsilon \right),$$

where *β*_*0*_ represents log of the overall mean prevalence rate, *β*_*1*_ represents the effect of a unit increase in risk factor *X*, and *η* represents a mean-zero Gaussian process accounting for spatial correlation induced by additional dependence not captured by *X*. The spatially correlated error term *η* follows a multivariate normal distribution with a variance-covariance matrix ***C***, in which each entry *c*_*ij*_ represents the covariance between the residuals at the *i*th and the *j*th location when *i* ≠ *j*, and the variance of the residuals at the *i*th location when *i* = *j*. Covariance between sites *i* and *j* is specified as $c_{ij}=\sigma_{s}^{2}e^{-d_{ij}/\rho }$, where *d*_*ij*_ is the distance between sites *i* and *j*, and *ρ* is the scale parameter as before; and the variance at site *i* is *σ*_*d*_^*2*^ + *σ*_*s*_^*2*^. The correlation of the residuals between two sites as a function of the distance between them is shown in S2 Fig. Parameters *β*_*0*_, *β*_*1*_, *σ*_*s*_, *σ*_*d*_, and *ρ* were estimated based on the prevalence rates and risk factor levels at the 30 in-network sites, after which 1000 realizations of log-prevalence rates at the 70 candidate sites were drawn according to the fitted parameters, observed prevalence at in-network sites, risk factor levels at candidate sites, and distance matrix between in-network and candidate sites.

#### Surveillance model specification

Relevant aspects of information captured by the surveillance system in this demonstration pertain to the extrapolation of prevalence from enrolled to unenrolled sites, as well as the variance of the estimated effect of risk factor *X*. Assuming perfect enumeration of disease prevalence at each enrolled site, as well as known values of the risk factor *X* for all sites, information drawn by each candidate design is represented by improvements in estimates of *β*_1_ and predictions at 70-n out-of-network sites obtained by fitting a universal kriging predictor to data from enrolled sites [51]. In this demonstration, we specify the spatial covariance structure of the true disease process and assume it is known to the surveillance system operator during optimization. However, we note that real world users of DIOS would ideally obtain this information by validating the disease model against surveillance data so as to select a well-supported model structure. If insufficient data are available to indicate a valid structure, several alternative approaches could be considered. One could simulate a distribution of performance metrics for each design using an ensemble of plausible disease models, and return all designs achieving a target probability of being included in the Pareto-optimal set. Another approach might be to define objective functions on the basis of some proximal measure related to performance metrics of interest for which model uncertainty is less of a concern, e.g., minimizing the average distance between the unmonitored and monitored locations, which promotes uniform coverage of the study region. Another potential remedy would be to undertake additional data collection using simple random sampling or grid sampling before performing simulation-optimization using the DIOS framework.

#### Design space

In our hypothetical example, we have an existing network of 30 surveillance sites {*s*_1_ … *s*_30_}, and 70 additional locations {*s*_31_ … *s*_100_} from which we may select *n* new sites to be added to the network. Therefore, our design parameter *s*_*θ*_ is the set of *n* sites to be added to the network, and the discrete design space is all possible sets of *n* sites chosen from 70.

### Optimization

#### Objective functions: Spatial interpolation

The first surveillance function we wish to optimize is prediction of the geographical distribution of the disease. Therefore, we define the objective function as the mean squared error (MSE) of log predicted prevalence at the *70-n* out-of-network locations after adding *s*_*θ*_ to the network:

$$f_{1}(s_{\theta })=\sum_{k=1}^{1000}\sum_{j=1}^{70-n}{\left(Y_{d_{k,j}}-{\hat{Y}}_{d_{k,j}}(s_{\theta })\right)}^{2}/((70-n)*1000),$$

where $Y_{d_{k,j}}$ represents the log prevalence rate at the *j*th unenrolled site in the *k*th disease system model realization, while ${\hat{Y}}_{d_{k,j}}(s_{\theta })$ represents the predicted log prevalence rate at the *j*th site after augmenting the existing network with *s*_*θ*_ in the *k*th realization. We denote the objective function value for this objective as OFV1. Other objective functions, such as the MSE of log predicted prevalence rate at the existing 30 sites or across all 100 sites, can also be used. Existing literature on optimal spatial design provides more options for relevant objective functions [52–54].

#### Objective functions: Effect estimation

Our second surveillance goal is precise estimation of the effect of the risk factor *X* on the disease outcome, so the objective function is formalized as:

$$f_{2}(s_{\theta })=\sum_{k=1}^{1000}var({\hat{\beta_{1}}}_{d_{k}}(s_{\theta }))/1000,$$

where ${\hat{\beta_{1}}}_{d_{k}}(s_{\theta })$ represents the estimate of *β*_*1*_ after augmenting the existing network with *s*_*θ*_ in the *k*th disease system model realization.

#### Search algorithms

When a single site is to be added to the network, the design space is small enough to allow for evaluation of the objective function across all possible designs. Therefore, the algorithm for proposing new designs simply steps sequentially through sites {*s*_31_ … *s*_100_}. However, when the optimization question is shifted to the best three sites to add, the design space expands to 54,740 combinations, making sequential enumeration a prohibitively expensive search strategy. Under these conditions, heuristic (greedy) or metaheuristic algorithms play an important role in finding the optimal or near-optimal solution within a reasonable amount of time [55]. Moreover, the evaluation of the objective function across realizations can be parallelized to further reduce computational time.

We illustrate the use of a simulated annealing (SA) meta-heuristic algorithm popular in spatial sampling network design [56,57] to more efficiently explore the design space when three sites are to be added. In SA, a random initial design is proposed, after which, at each iteration, a new design is sampled from the neighborhood of the current design and the objective function value (OFV) for the new design is evaluated. Here, the neighborhood of a set of *n* sites to enroll is defined as designs sharing *n-1* sites with the current design. If the new OFV is superior to the current OFV, the new design is accepted with 100% probability; otherwise, it is accepted with a probability of $e^{-\frac{\Delta OFV}{T}}$, where Δ*OFV* is the change in the OFV and *T* is a tuning parameter analogous to temperature [58]. *T* decreases at a rate *α* after each iteration, causing SA to accept deterioration in the OFV more frequently at the beginning of the run and rarely towards the end. Probabilistically accepting worse solutions early in the search enables the algorithm to escape local optima. For our demonstration, we set the initial temperature *T*_*0*_ and cooling rate *α* separately for each objective and epidemiologic scenario, following suggestions by Sait and Youssef [58], and set the stopping criteria is to be *T≤10*^*−6*^. We repeat the SA process 3 times to examine the convergence of the result.

### Optimal surveillance designs

#### Selecting one additional site to optimize spatial prediction

**Fig 3:**
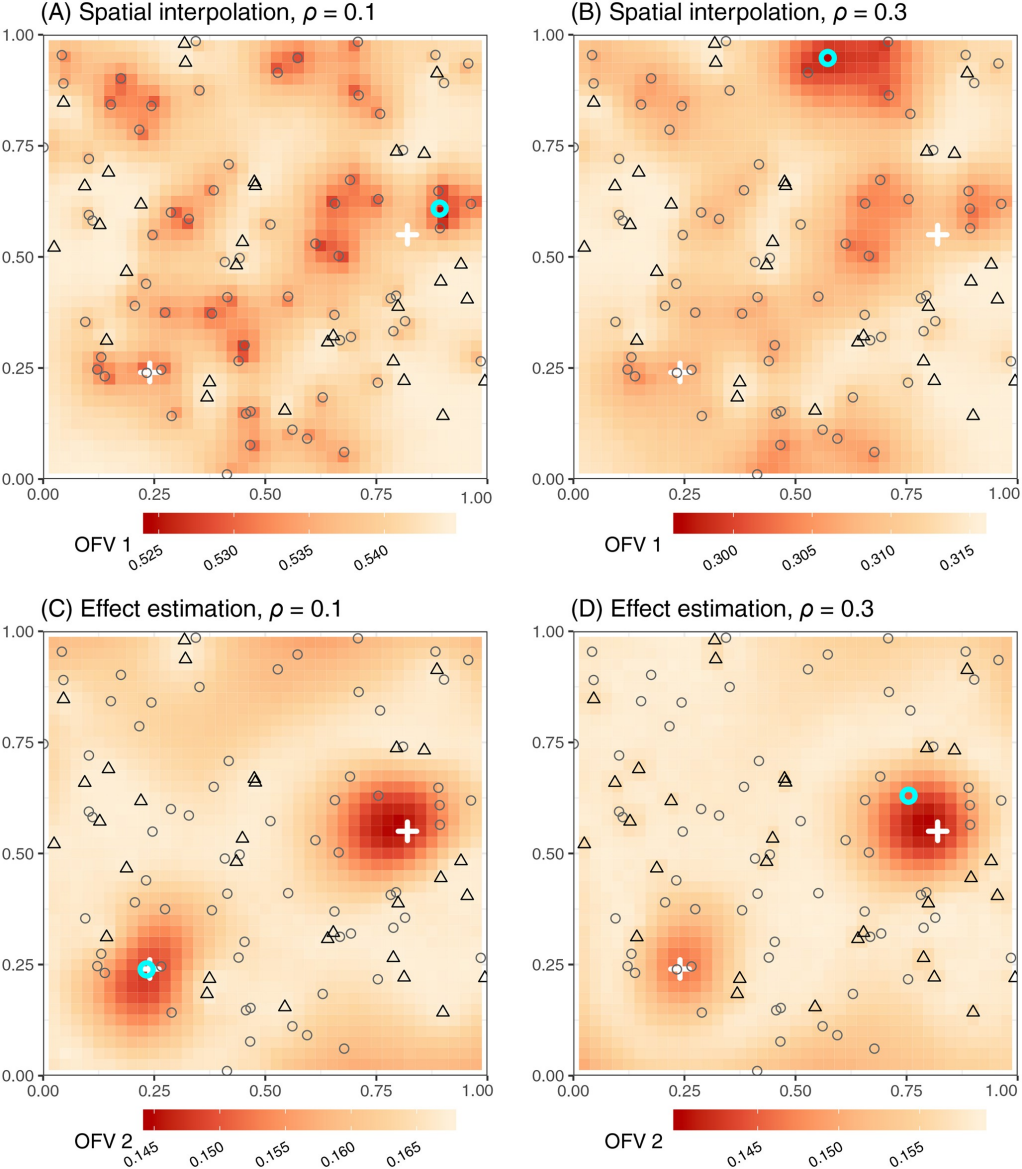
Optimal site placement to augment a surveillance network for spatial prediction or effect estimation under scenarios of spatially patchy or smooth disease distributions. Black triangles represent initially enrolled sites, gray circles represent unselected candidate sites, and the cyan circle indicates the optimal site to add to the network. White crosses represent point sources for risk factor *X*. Raster colors represent objective function values for hypothetical sites added across a regular 41*41 grid in order to visualize the response surface in relation to initial network locations and the underlying risk factor. Colors represent the mean squared error of spatial predictions at unmonitored sites in A and B, and the variance of effect estimation for risk factor *X* in C and D.

The mean squared error of spatial predictions across unenrolled sites (OFV1) is minimized by enrolling sites that are in close proximity to multiple out-of-network sites—especially clusters of unmeasured sites at long distances from existing network locations (Fig 3A and 3B). These optimal placements address informational gaps by enrolling sites that increase the average covariance between measured and unmeasured locations, and tend to fall in areas close to several unenrolled sites but away from the initially enrolled locations. Furthermore, the amount of information that can be inferred from the same set of neighboring sites increases with the scale parameter *ρ*. Thus, in the spatially patchy disease scenario, where the scale of spatial autocorrelation is small, optimal placement occurs in the center of a tight cluster of unenrolled sites (Fig 3A). Under the spatially smooth scenario, the same cluster is correlated with initially enrolled sites, and optimal site placement falls in the center of a loose cluster of unmeasured sites located quite far from the initial network (Fig 3B). Under the parameter set used to generate demonstration data, there is no clear influence of risk factor level *X* on site selection to optimize spatial prediction.

#### Selecting one additional site to optimize effect estimation

The variance of the estimated effect of risk factor *X* on log disease prevalence (OFV2) is lower when values of *X* at added sites lie towards an extreme of *X*’s observed range and when the site to be added is relatively uncorrelated with (i.e., distant from) initially enrolled sites (Fig 3C and 3D). In the spatially patchy disease scenario, where the scale of spatial autocorrelation is limited, optimal site placement is dominated by the level of risk factor *X*, and the available site with highest *X* is chosen (Fig 3C). In the spatially smooth scenario, which has an extended scale of spatial autocorrelation, the correlation of outcomes between the site with the highest *X* and nearby initially enrolled sites results in selection of an alternative location where *X* is lower, but prevalence is expected to be more independent of previously observed outcomes (Fig 3D).

#### Single site selection based on multiple objectives

**Fig 4:**
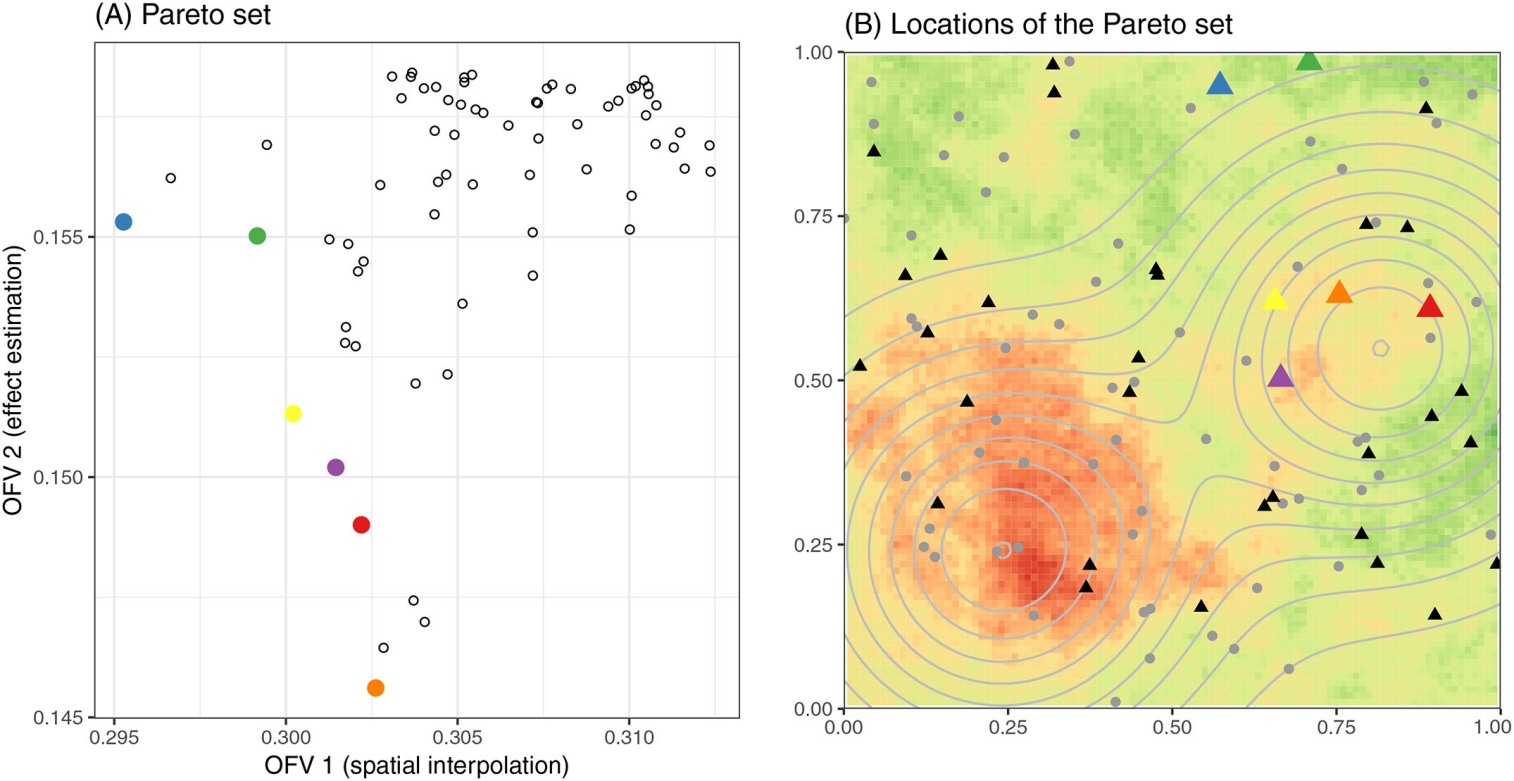
Results from Pareto optimization under the spatially smooth disease scenario (*ρ* = 0.3). (A) Mean squared error of log predicted disease prevalence (OFV1) and variance of causal effect estimate (OFV2) of the Pareto set (colored dots) and all other candidate sites (hollow dots). (B) Locations of the Pareto set (colored triangles) colored coded as in Panel A. Black triangles represent initially enrolled sites, and gray dots represent unchosen candidate sites. Background color in Panel B represents log prevalence when *ρ* = 0.3 using the same color scheme as in Fig 2C, while contour lines represent levels of risk factor *X*.

When simultaneously optimizing site enrollment for spatial prediction and effect estimation, the output is a Pareto optimal set containing designs that are considered equally optimal because no objective function value can be improved without impairing the other objective function values. A set of six candidate sites emerges for the spatially smooth disease scenario, including four alternative selections to the optimal locations for each single objective (Fig 4). The Pareto optimal set for the spatially patchy scenario includes only one non-dominated site in addition to the optimal locations for either objective individually (S3 Fig). Since Pareto optimization does not return a single solution, some way of reconciling the objective criteria, such as a weighted sum or expression of total cost may be required to choose the optimal design. Notably, we did not incorporate cost associated with adding sites in our analysis, but this could be accomplished by including cost or number of sites as an objective function to be minimized, and modifying the SA algorithm to allow adding, dropping, or swapping sites when finding neighboring designs. In this case, the spatial prediction OFV, effect estimation OFV, and the cost-effectiveness OFV would be jointly optimized.

#### Selecting three additional sites to optimize spatial prediction

**Fig 5:**
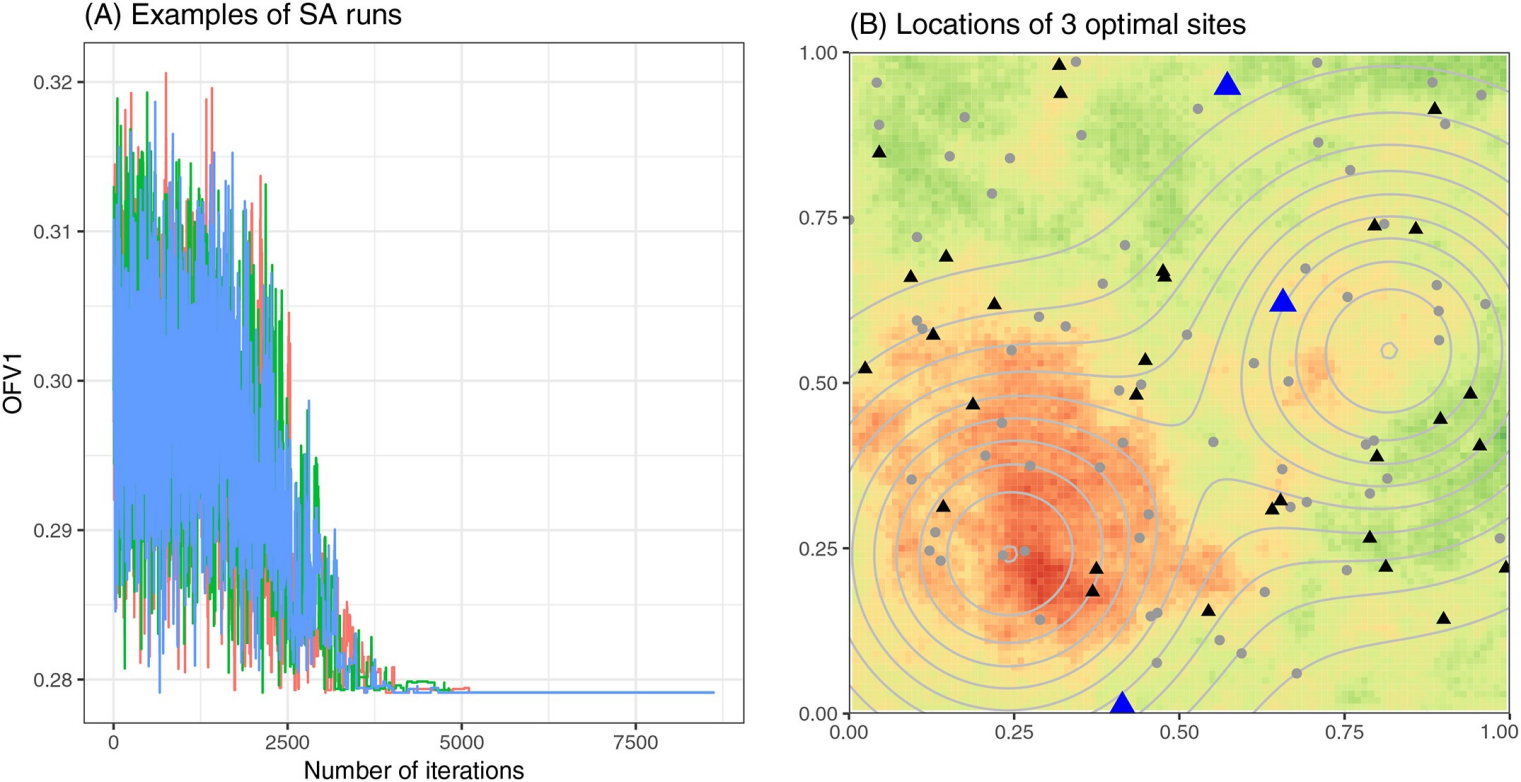
Metaheuristic optimization with simulated annealing (spatial prediction, *ρ* = 0.3). (A) Mean squared error of predicted log prevalence (OFV1) across iterations of three SA runs. (B) The locations of the optimal 3 sites. Black triangles represent existing sites, blue triangles represent the optimal additional sites, and gray dots represent unchosen alternative sites. Background color in Panel B represents log prevalence when *ρ* = 0.3 using the same color scheme as in Fig 2C, while contour lines represent levels of risk factor X.

As a final example, we demonstrate the use of metaheuristic algorithms to search larger design spaces, applying simulated annealing to select three additional sites out of seventy candidate sites simultaneously. Simulated annealing optimizations seeded with different initial designs converged to the same best set of three additional sites to enroll for enhanced spatial prediction under the spatially smooth disease scenario (Fig 5). All three SA runs (Fig 5A, colored lines) converged to the same optimal design within 6,000 iterations. Given the parameters and the stopping criteria we used, each run terminated after 8,630 iterations. Even with three runs, the total number of objective function evaluations was 25,890, less than half of what would be required if using enumeration. Fig 5B shows the location of the optimal three-site set. The results for spatial prediction under the spatially patchy outcome scenario, as well as for effect estimation under both the spatially patchy and smooth outcome scenario, are shown in S4–S6 Figs. Computational run times for these scenarios are shown in S1 Table.

## Conclusion

Surveillance system designs that provide reliable, timely estimates of the spatio-temporal distributions of endemic and epidemic diseases, are critical to the efficient allocation of resources for public health responses. However, opportunities to apply numerical optimization to surveillance system design have heretofore been overlooked in the literature. In this paper, we presented and provided a basic demonstration of DIOS, a framework for surveillance optimization via simulation to enhance design decision making and facilitate research into optimal design principles under uncertain or changing epidemiological conditions.

The scope of surveillance objectives, design parameters, and contexts extends far beyond the demonstration provided in this paper, which applied the DIOS framework to a specific spatial design optimization problem. DIOS is suitable for application to a wide range of surveillance design problems (Table 1). In real-world applications, it may be prudent to accommodate structural uncertainties via an ensemble of plausible disease and/or surveillance models in order to ensure that optimization output is not biased by unverifiable assumptions regarding the unobservable ‘true’ state of the disease system. Furthermore, cost and efficiency considerations, represented only abstractly as absolute limits on the number of new monitoring locations in our demonstration, are likely to be of major concern in practical applications of the DIOS framework. There are numerous ways in which such operational considerations can be incorporated into DIOS, encompassing hard constraints on design parameters, and, potentially, conducting performance optimization at various discrete levels of constraint to assess marginal benefits of additional investment. Penalty functions used to adjust separate measures of performance (e.g., information gain per dollar spent) or incorporation of measures of cost or effort as separate objectives to be minimized are additional possibilities. Our representation of design constraints was also simplified in that all candidate sites were considered equal with respect to surveillance quality and cost of enrollment. In reality, DIOS users may wish to incorporate prior information on performance into the surveillance model (e.g., simulating low random error associated with data originating from specific sites that have enhanced data collection or reporting infrastructure). Cost or effort associated with enrolling each site can be incorporated into the optimization procedure as constraints on design parameters, penalties in objective functions, or separate objectives that represent site preference.

The DIOS framework facilitates improved surveillance system designs by providing a quantitative platform for incorporation of data and theory on epidemiologic and surveillance processes in the context of specific surveillance objectives and resource and operational constraints. Our hope is that DIOS will stimulate collaboration between health planners, clinical care providers and laboratories, researchers, and software developers to advance understanding of surveillance design under uncertainty, and indeed, such collaborations will be crucial to its utility for practical applications. Input from public health professionals is needed to specify proper objective functions and relevant design parameters, select meaningful constraints on design parameters, construct surveillance system models that accurately represent real-world surveillance processes, provide information on operational or logistical design constraints and minimal acceptable performance on various objectives, and to provide preference information to guide selection of the “best” surveillance design either during or after optimization (e.g., human-in-the-loop methods [59] or selection from Pareto-optimal designs, respectively). The rationality of the output optimal design will be highly dependent on the accuracy and relevance of data or models used to represent disease and surveillance processes during optimization, as well as the performance of the optimization search algorithm. There is much future work to be done to develop and validate simulation models that can represent relevant epidemiologic and measurement processes accurately; to analyze the sensitivity of optimal design to the specification of disease system models and changes in disease epidemiology; and to adopt optimization approaches from related fields—such as environmental monitoring network design and signal processing [60–62]—to disease surveillance design applications. Lastly, while our discussion and framing has focused on surveillance infrastructures and objectives related to measuring the incidence of human disease, we note that DIOS can also be applied to the optimization of surveillance information informed by other data streams, such as vector or environmental surveillance. There are indeed many exciting and relevant questions surrounding optimal design and integration of these newer types of surveillance with human incidence data which DIOS may help to address.

## Supporting information

S1 Text Data simulation methods. (DOCX)

S1 Fig Five realizations of the log prevalence surface when ρ = 0.1 (left panels) or 0.3 (right panels). (PDF)

S2 Fig Correlation as a function of distance between locations when ρ = 0.1 (solid curve) or 0.3 (dashed curve). (PDF)

S3 Fig Results from Pareto optimization (ρ = 0.1). (A) OFV1 and OFV2 of the Pareto set (colored dots) and all other each candidate site (hollow dots). (B) Spatial locations of the Pareto set (colored triangles) colored by the same color scheme as in Panel A. Black triangles represent existing sites, and gray dots represent unchosen alternative sites. Background color represents log prevalence value when ρ = 0.1 using the same color scheme as in Fig 2B, while contour lines represent levels of risk factor X.
(PDF)

S4 Fig Iterative optimization with simulated annealing (spatial interpolation, ρ = 0.1). (A) OFV1 against the number of iterations in 3 SA runs. (B) The locations of the optimal 3 sites. Black triangles represent existing sites, blue triangles represent the optimal additional sites, and gray dots represent unchosen alternative sites. Background color represents log prevalence value when ρ = 0.1 using the same color scheme as in Fig 2B, while contour lines represent levels of risk factor X.
(PDF)

S5 Fig Iterative optimization with simulated annealing (effect estimation, ρ = 0.1). (A) OFV1 against the number of iterations in 3 SA runs. (B) The locations of the optimal 3 sites. Black triangles represent existing sites, blue triangles represent the optimal additional sites, and gray dots represent unchosen alternative sites. Background color represents log prevalence value when ρ = 0.1 using the same color scheme as in Fig 2B, while contour lines represent levels of risk factor X.
(PDF)

S6 Fig Iterative optimization with simulated annealing (effect estimation, ρ = 0.3). (A) OFV1 against the number of iterations in 3 SA runs. (B) The locations of the optimal 3 sites. Black triangles represent existing sites, blue triangles represent the optimal additional sites, and gray dots represent unchosen alternative sites. Background color represents log prevalence value when ρ = 0.3 using the same color scheme as in Fig 2C, while contour lines represent levels of risk factor X.
(PDF)

S1 Table Computational run time for selecting three additional sites with simulated annealing on a node with 64 GB of RAM and two Intel Xeon 12-core 2.6 GHz Haswell processors. (XLSX)
