# Supplementary material for: The DIOS framework for optimizing infectious disease surveillance: Numerical methods for simulation and multi-objective optimization of surveillance network architectures
Source: PLoS Comput Biol. 2020 Dec 4;16(12):e1008477. doi: 10.1371/journal.pcbi.1008477 (PMC7744064; doi:10.1371/journal.pcbi.1008477)
Supplement: S1 Text — (DOCX) [file pcbi.1008477.s001.docx]

**Supplementary Text 1: Data simulation methods**

**Other input data.** A risk factor *X* that emanates from two point sources, such as the locations of mass gatherings or factories that emit air pollution, was generated. The level of the risk factor at *s* is determined by $X\left( s \right)= \frac{1}{\sqrt{2\pi}\sigma}e^{-\frac{d_{s, s_{p1}}}{2\sigma^{2}}}+\frac{1}{\sqrt{2\pi}\sigma}e^{-\frac{d_{s, s_{p2}}}{2\sigma^{2}}}$, with σ equals 0.2 and $d_{s, s_{pi}}$represents the distance between s and the *i*th point source.

**Epidemiologic data.** We simulate disease prevalence, $Y$, for 100 random locations within a unit square based on the known spatial distribution of a causal risk factor, $X$, and under two distinct scenarios of spatial autocorrelation in disease outcomes. The log prevalence for these sites is generated as:

$Y=\exp\left( \beta_{0}+\beta_{1}X+\eta+\varepsilon\right)$ (1)

where *β_0_* represents the overall log mean prevalence, *β_1_* represents the effect of a unit increase in risk factor $X$, *η* represents a mean-zero Gaussian process accounting for the spatial correlation contributed by unmeasured risk factors, and *ε* represents independent and identically distributed mean-zero normally distributed noise with a variance of *σ_d_^2^*. The spatially correlated error term *η* can be represented by a multivariate normal distribution with a variance-covariance matrix **C**, in which each entry c_ij_ represents the covariance between the residuals at the *i*th and the *j*th location when *i* ≠ *j*, and the spatial variance *σ_s_^2^* when *i* = *j*. We simulate our disease system to be consistent with the widely used second-order stationary assumption (the covariance between the residuals at two locations only depends on the distance between them) and exponential covariance function in specifying **C**, so the covariance between sites i and j can be written as $\text{c}_{\text{ij}}\text{= }\text{σ}\text{s}\text{2}\text{e}^{\text{-}\text{d}_{\text{ij}}\text{/ρ}}$. The spatial range parameter, [
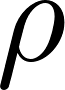
](https://www.codecogs.com/eqnedit.php?latex=%5Crho%250), defines the range of spatial autocorrelation, and it is this parameter that we vary to obtain scenarios of relatively smooth ($\rho=0.3$) and patchy ($\rho=0.1$) spatial variation in prevalence unexplained by $X$. The parameters *β_0_*, *β_1_*, *σ_s_*, and *σ_d_* are set to be -8.5, 1, 1, and 0.1, respectively. Only the disease data at a randomly sampled 30 in-network sites {$s_{1}\ldots s_{30}$} are available to the designer. Data at the other 70 unmonitored locations $\left\{ s_{31}\ldots s_{100} \right\}$ are treated as unknown.
