# Supplementary figures and images for: The DIOS framework for optimizing infectious disease surveillance: Numerical methods for simulation and multi-objective optimization of surveillance network architectures
Source: PLoS Comput Biol. 2020 Dec 4;16(12):e1008477. doi: 10.1371/journal.pcbi.1008477 (PMC7744064; doi:10.1371/journal.pcbi.1008477)

$\rho = 0.1$

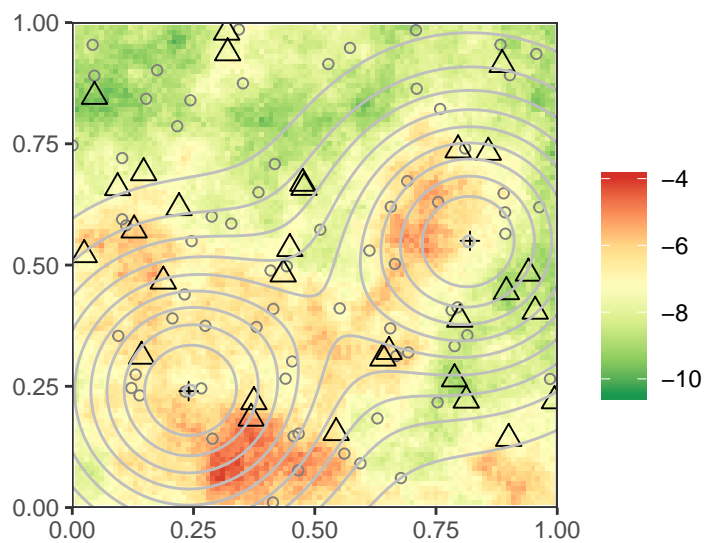

$\rho = 0.3$

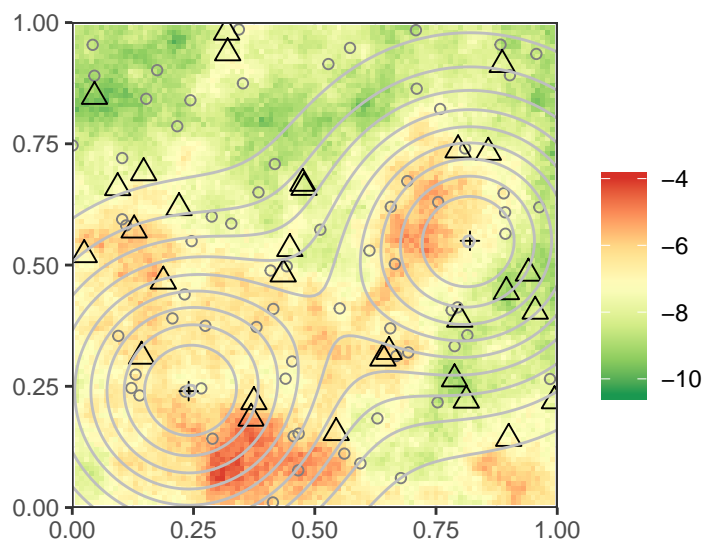

$\rho = 0.1$

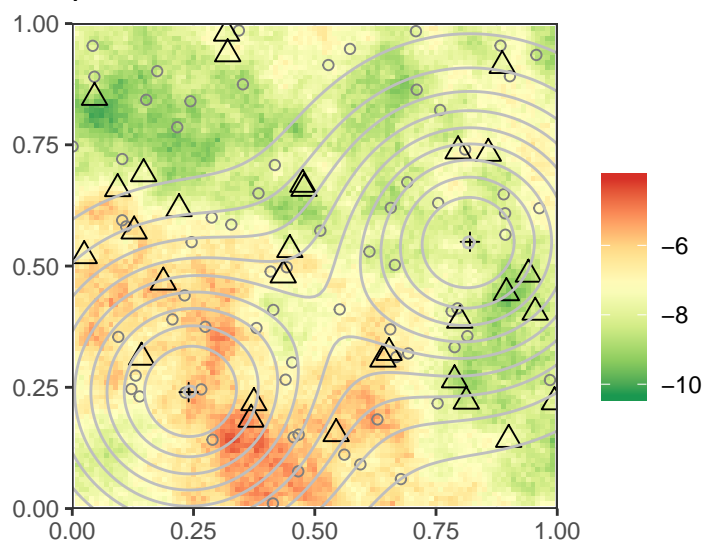

$\rho = 0.3$

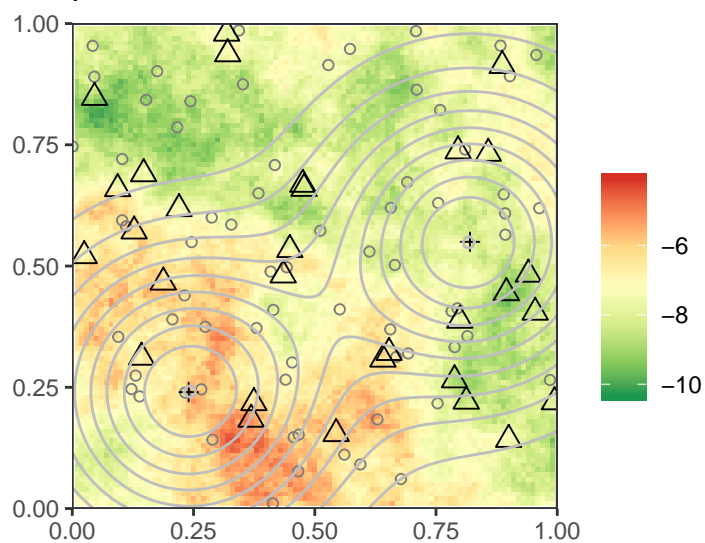

$\rho = 0.1$

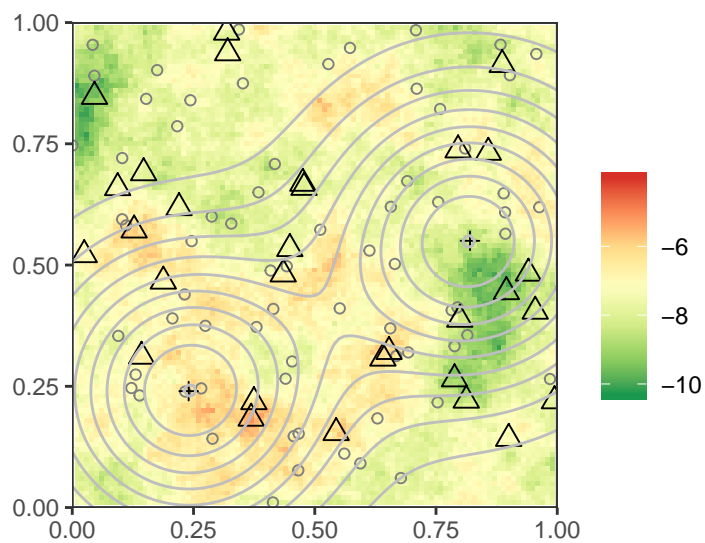

$\rho = 0.3$

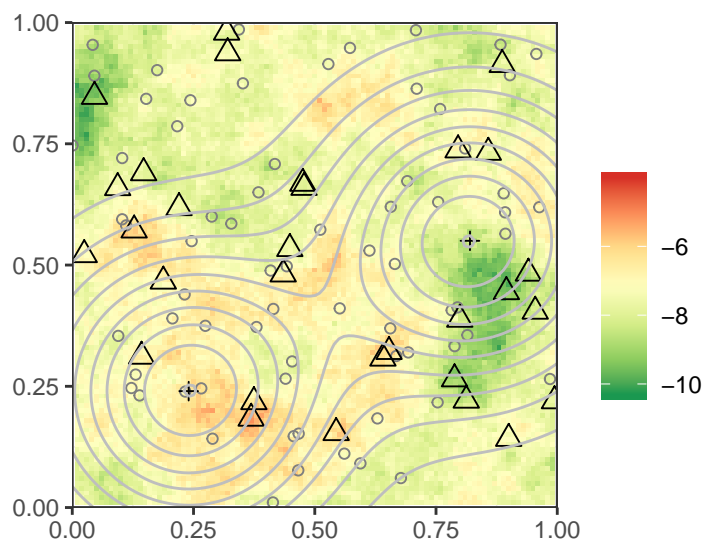

Supplement: S1 Fig — (PDF) [file pcbi.1008477.s002.pdf]

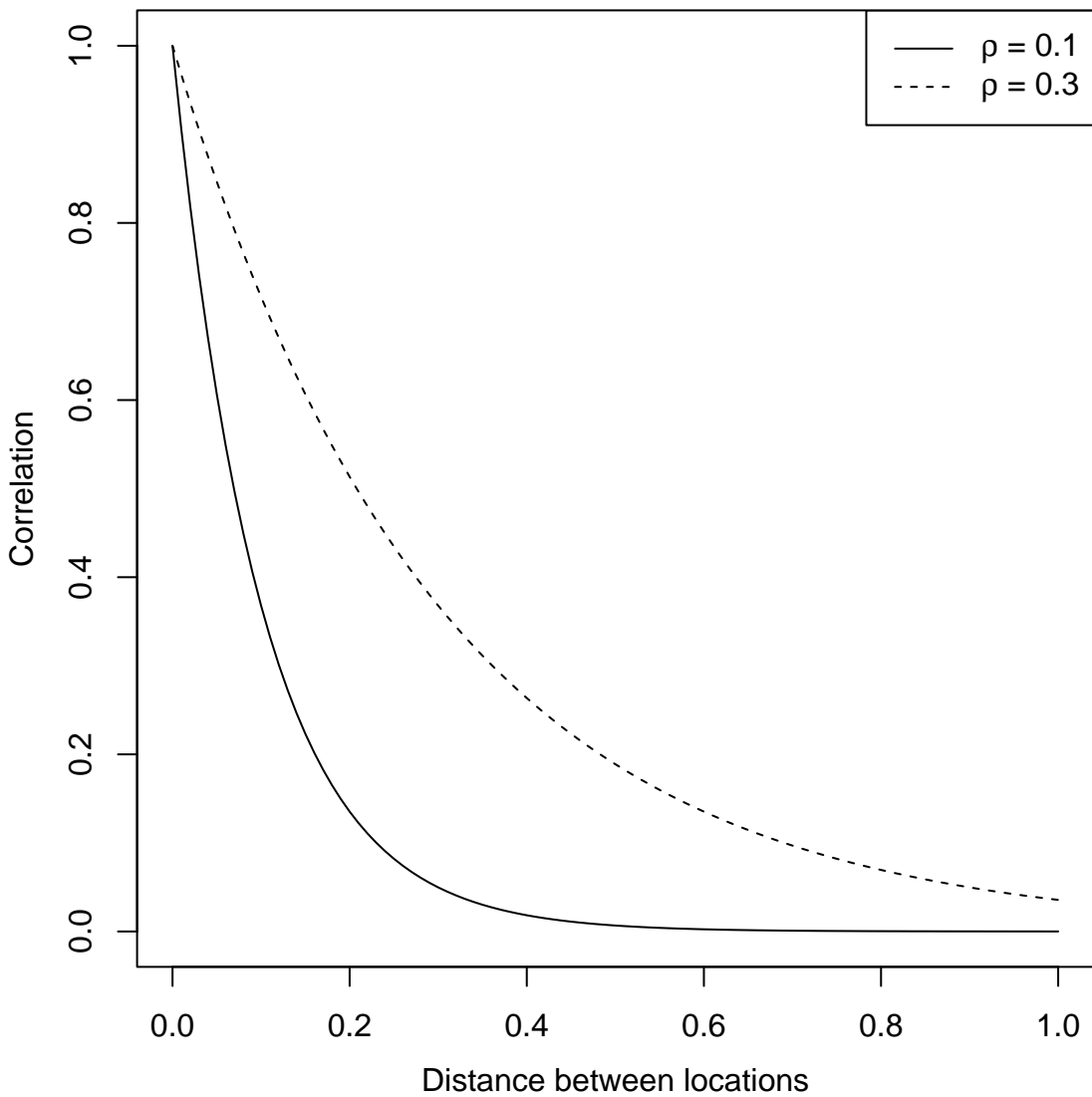

Supplement: S2 Fig — (PDF) [file pcbi.1008477.s003.pdf]

(A) Pareto set

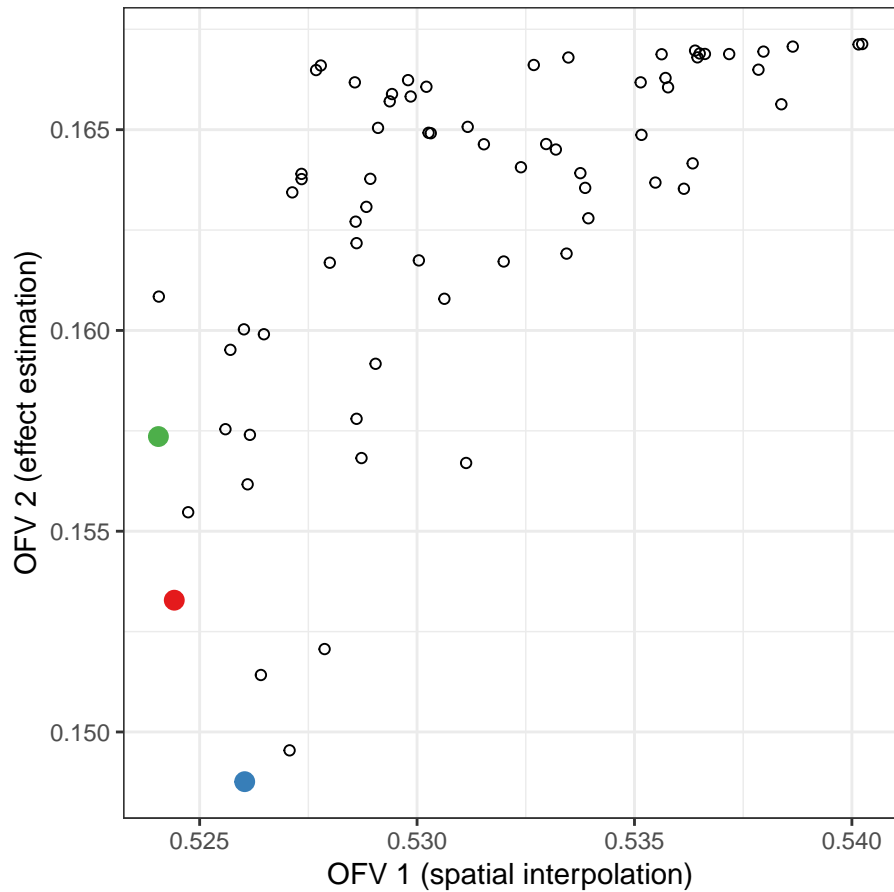

(B) Locations of the Pareto set

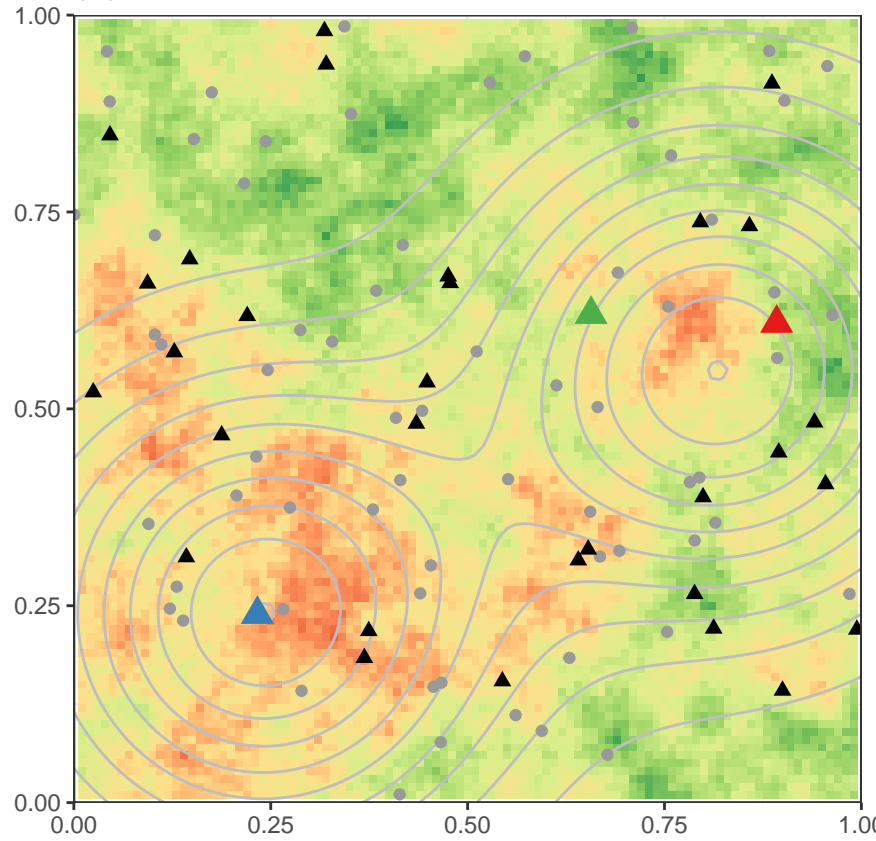

Supplement: S3 Fig — (A) OFV1 and OFV2 of the Pareto set (colored dots) and all other each candidate site (hollow dots). (B) Spatial locations of the Pareto set (colored triangles) colored by the same color scheme as in Panel A. Black triangles represent existing sites, and gray dots represent unchosen alternative sites. Background color represents log prevalence value when ρ = 0.1 using the same color scheme as in Fig 2B, while contour lines represent levels of risk factor X. (PDF) [file pcbi.1008477.s004.pdf]

(A) Examples of SA runs

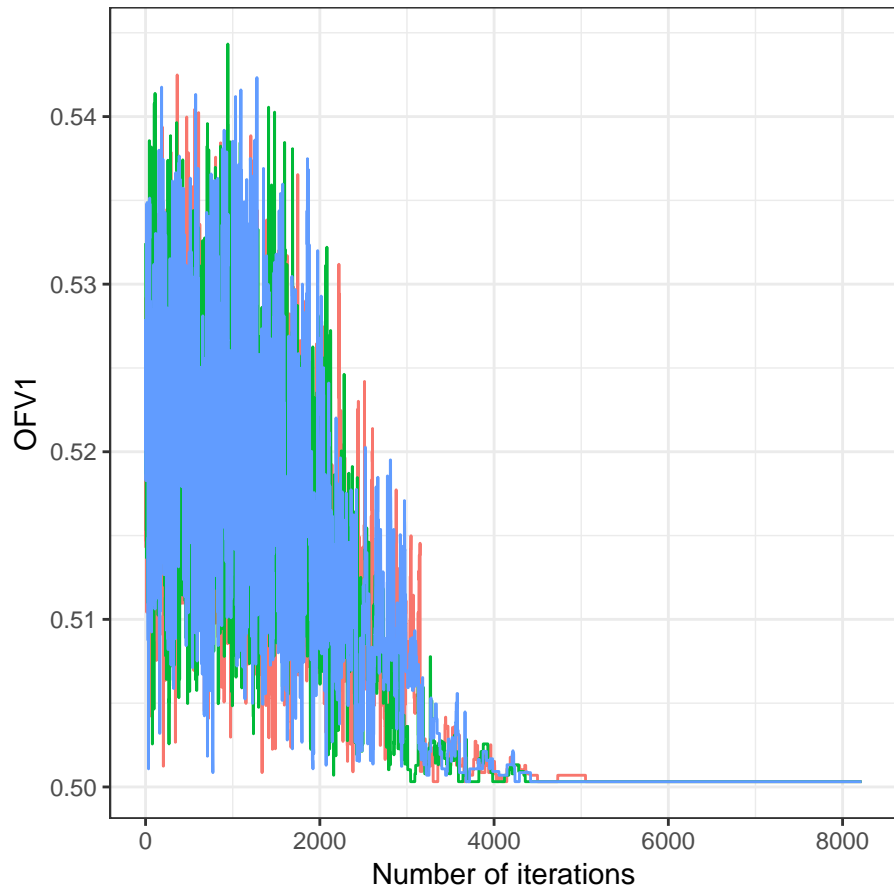

(B) Locations of 3 optimal sites

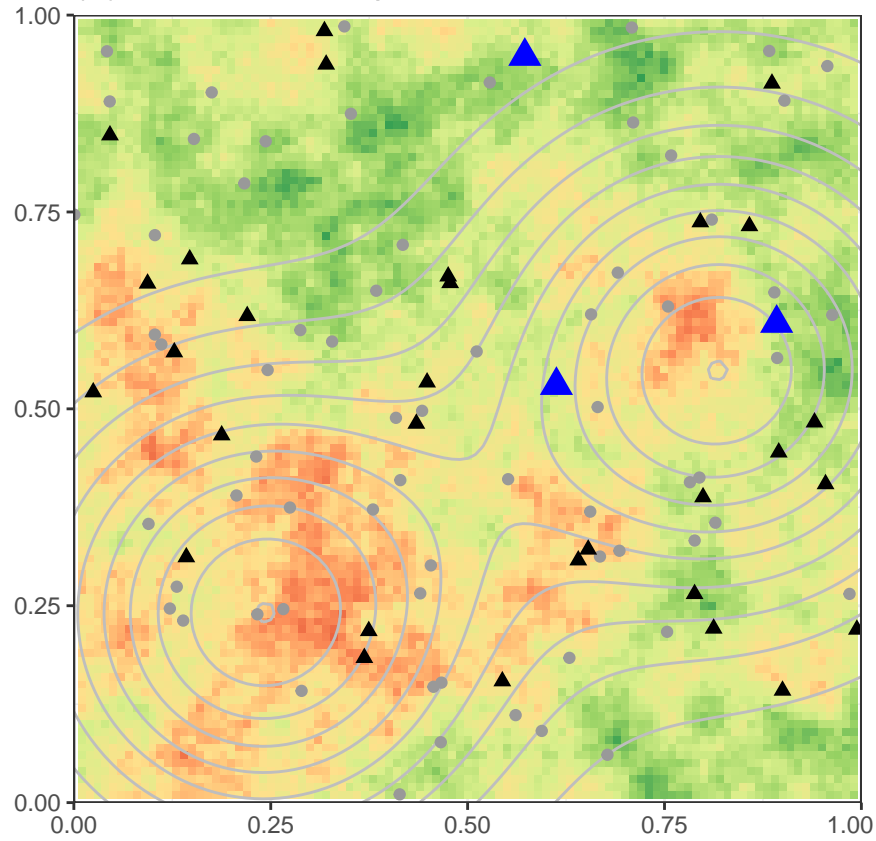

Supplement: S4 Fig — (A) OFV1 against the number of iterations in 3 SA runs. (B) The locations of the optimal 3 sites. Black triangles represent existing sites, blue triangles represent the optimal additional sites, and gray dots represent unchosen alternative sites. Background color represents log prevalence value when ρ = 0.1 using the same color scheme as in Fig 2B, while contour lines represent levels of risk factor X. (PDF) [file pcbi.1008477.s005.pdf]

(A) Examples of SA runs

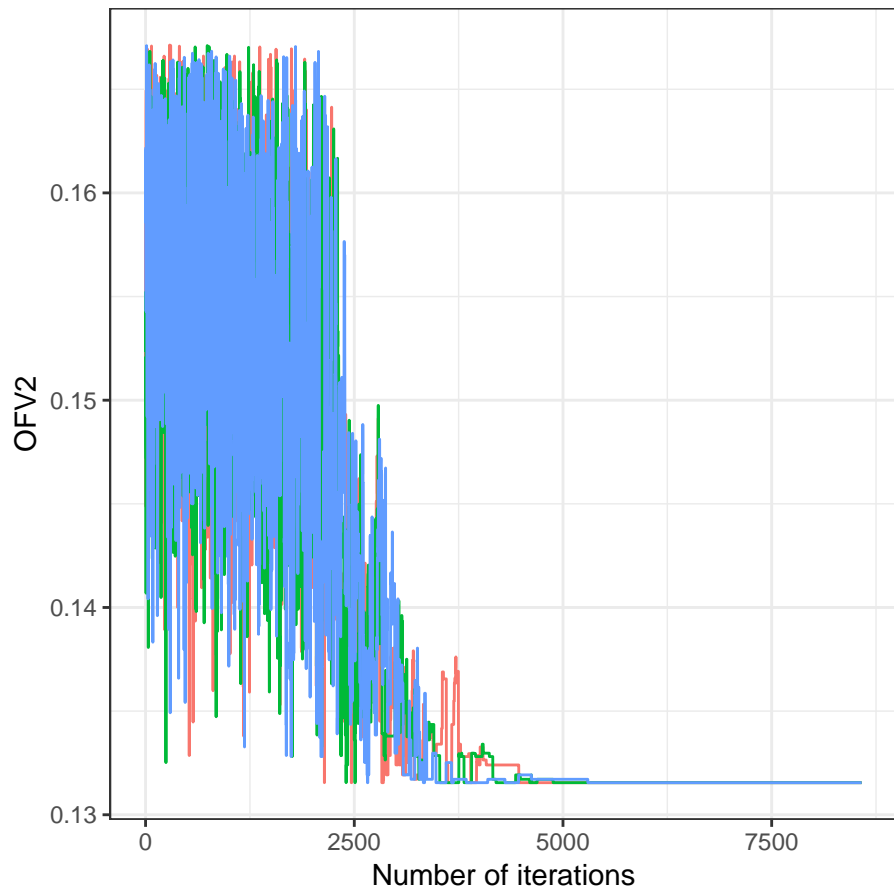

(B) Locations of 3 optimal sites

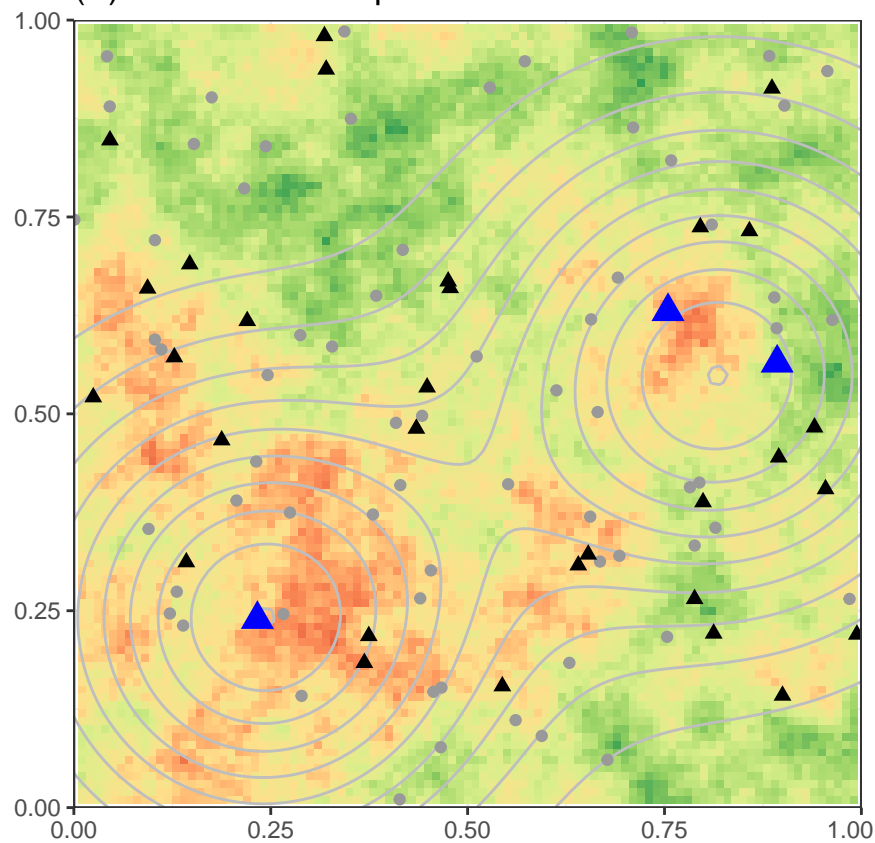

Supplement: S5 Fig — (A) OFV1 against the number of iterations in 3 SA runs. (B) The locations of the optimal 3 sites. Black triangles represent existing sites, blue triangles represent the optimal additional sites, and gray dots represent unchosen alternative sites. Background color represents log prevalence value when ρ = 0.1 using the same color scheme as in Fig 2B, while contour lines represent levels of risk factor X. (PDF) [file pcbi.1008477.s006.pdf]

(A) Examples of SA runs

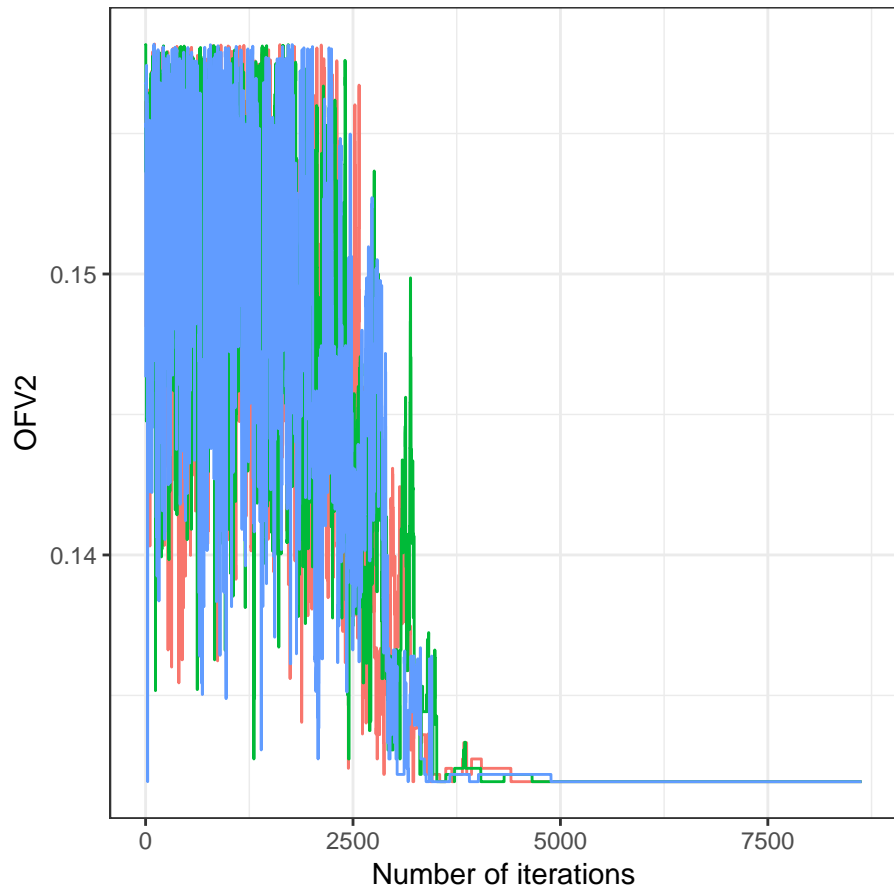

(B) Locations of 3 optimal sites

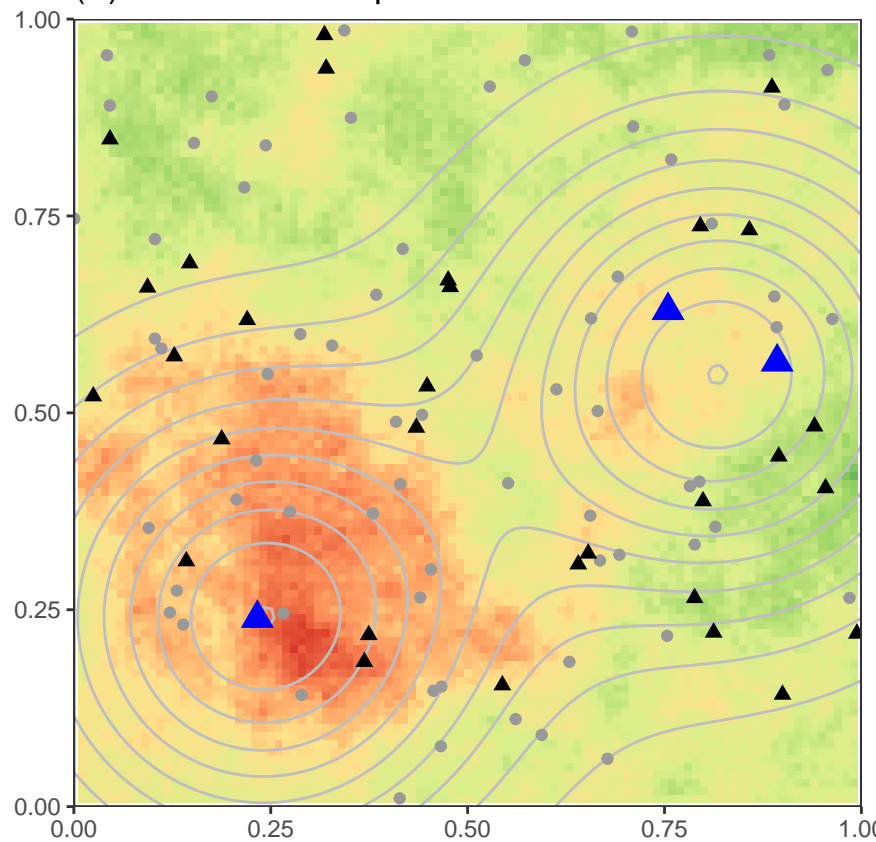

Supplement: S6 Fig — (A) OFV1 against the number of iterations in 3 SA runs. (B) The locations of the optimal 3 sites. Black triangles represent existing sites, blue triangles represent the optimal additional sites, and gray dots represent unchosen alternative sites. Background color represents log prevalence value when ρ = 0.3 using the same color scheme as in Fig 2C, while contour lines represent levels of risk factor X. (PDF) [file pcbi.1008477.s007.pdf]
